# Supplementary material for: Exceptional catalytic activity of oxygen evolution reaction via two-dimensional graphene multilayer confined metal-organic frameworks
Source: Nat Commun. 2022 Oct 18;13:6171. doi: 10.1038/s41467-022-33847-z (PMC9579180; doi:10.1038/s41467-022-33847-z)
Supplement: Supplementary file 1 — Supplementary Information [file 41467_2022_33847_MOESM1_ESM.pdf]

Supplementary Information for

Exceptional Catalytic Activity of Oxygen Evolution Reaction via Two-Dimensional Graphene Multilayer Confined Metal-Organic Frameworks

Siliu Lyu[1,2#], Chenxi Guo[3#], Jianing Wang[2], Zhongjian Li[1,4], Bin Yang[1,4], Lecheng Lei[1,4], Liping Wang[2,5], Jianping Xiao[3,5,6]*, Tao Zhang[2,5]*, Yang Hou[1,4]*

[1]Key Laboratory of Biomass Chemical Engineering of Ministry of Education, College of Chemical and Biological Engineering, Zhejiang University, 38 Zheda Road, Hangzhou 310027, China.

[2]Ningbo Institute of Materials Technology and Engineering, Chinese Academy of Sciences, 1219 Zhongguan West Road, Ningbo 315201, China.

[3]State Key Laboratory of Catalysis, Dalian Institute of Chemical Physics, Chinese Academy of Sciences, Dalian National Laboratory for Clean Energy, 457 Zhongshan Road, Dalian 116023, China.

[4]Institute of Zhejiang University-Quzhou, 78 Jiuhua Boulevard North, Quzhou 324000, China.

[5]University of Chinese Academy of Sciences, Beijing, 100049, China.

[6]Dalian National Laboratory for Clean Energy, Dalian 116023, China.

#The authors have equal contributions in this work.

*Corresponding author. Email: xiao@dicp.ac.cn; tzhang@nimte.ac.cn; yhou@zju.edu.cn

Contents:

Supplementary Figs. 1-44

Supplementary Tables 1-13

Materials

1,3,5-Benzenetricarboxylic acid (95%, Sigma-Aldrich), 2,6-Naphthalenedicarboxylic acid dipotassium salt (95%, Sigma-Aldrich), Terephthalic acid (98%, Sigma-Aldrich), 1,2,3,4-Cyclobutanetetracarboxylic acid (≥98%, TCI), Nickel(II) chloride hexahydrate ($NiCl_6 \cdot 6H_2O$, 99.95%, Alfa Aesar), Nickel(II) acetate tetrahydrate ($Ni(CH_3COO)_2 \cdot 4H_2O$, ≥99.0%, Sigma-Aldrich), Iron(III) nitrate nonahydrate ($Fe(NO_3)_3 \cdot 9H_2O$, ≥98%, Sigma-Aldrich), Sodium hydroxide (NaOH, ≥98%, Alfa Aesar), Nafion (5% in lower aliphatic alcohols and water (15-20%), Sigma Aldrich), graphite foil (1 × 200 × 300 mm, Inner Mongolia carbon Co., Ltd), Ir/C (10 wt.%, FuelCellStore), Iridium(IV) oxide (IrO_2, 99%, Sigma-Aldrich), Ruthenium(IV) oxide (RuO_2, 99.9%, Alfa Aesar), and Platinum on carbon catalyst (Pt/C, 10 wt.% Pt, Sigma Aldrich) were used as received. All aqueous solutions were prepared with high-purity de-ionized water (DI-water, resistance 18 MΩ cm^{-1}).

Electrode preparation

Synthesis of NiFe-BTC//G-*X*. The commercial graphite foil was successively rinsed with acetone, ethanol, and DI-water under sonication for 20 min, and then dried in oven at 60 ºC for 2 h. A simple two-electrode system was employed for intercalation of graphite with ions and organic molecules, in which platinum foil and the commercial graphite foil were kept in parallel at a constant distance of 2.5 cm and placed as cathode and anode, respectively. Firstly, an electrochemical exfoliation process was conducted by anodization of graphite foil in dilute sulfuric acid solution (0.5 M, 50 mL) with using Pt foil (10 × 10 mm) as counter electrode and graphite foil (10 × 30 mm) as working electrode under 3 V for 30 min. The exfoliated graphite foil was rinsed with DI-water several times and dried in oven at 60 ºC. Secondly, 420 mg of 1,3,5-benzenetricarboxylic acid and 240 mg of NaOH were dissolved in DI-water (75 mL) and rigorously stirred for 30 min to obtain transparent solution. Then, the dilated graphite foil was put into the above solution and intercalated by 1,3,5-benzenetricarboxylic group under 5 V for different time. The as-obtained graphite foil was immersed into DI-water and slightly sonicated at an ultrasonic frequency of 20 KHz for 30 min to remove the excessive organic 1,3,5-benzenetricarboxylic trisodium salt on the surface

of graphite foil as well as small pieces of deciduous graphite flakes. Finally, 713 mg of $NiCl_2·6H_2O$ and 1.21 g of $Fe(NO_3)_3·9H_2O$ were dissolved in a mixed solvent of DI-water and ethanol (100 mL, V:V=1:1) and stirred for 20 min to form transparent inorganic salt solution. The treated graphite was immersed into the above solution for 24 h, during which the NiFe-BTC was *in situ* fabricated. The as-prepared electrode (denoted as NiFe-BTC//G-*X*, *X* represents different incremental electrochemical intercalation time of organic ligands) was then taken out, rinsed with copious DI-water and ethanol, dried at 60 °C overnight, and directly employed to trigger the anodic reaction of water splitting without carbonization treatment. The loading amount of NiFe-BTC//G-2h was tested for 20 times through weight difference method and the results were normalized to 7.3 mg cm^{-2}.

Synthesis of Ni-BTC//G-2h. The Ni-BTC//G-2h electrode was prepared by following procedure to NiFe-BTC//G-2h except that only 713 mg of $NiCl_2·6H_2O$ was used as the metal precursor (Supplementary Movie 1).

Synthesis of Fe-BTC//G-2h. The Fe-BTC//G-2h electrode was prepared by following procedure to NiFe-BTC//G-2h except that only 1.21 g of $Fe(NO_3)_3·9H_2O$ was used as the metal precursor.

Synthesis of NiFe-NDC//G. The synthesis of NiFe-NDC//G was similar with NiFe-BTC//G. Contrastingly, 250 mg of 2,6-naphthalenedicarboxylic acid dipotassium was dissolved in DI-water (75 mL) and rigorously stirred for 30 min to obtain the electrolyte to insert the organic ligands into the dilated graphite multilayers. The electrochemical intercalation time of organic ligands was set as 1 h. Subsequently, 485 mg of $Ni(CH_3COO)_2·4H_2O$ and 788 mg of $Fe(NO_3)_3·9H_2O$ were dissolved in a mixed solvent of DI-water and ethanol (100 mL, V:V=1:1) and stirred for 20 min to form transparent inorganic salt solution. The treated graphite was immersed into the above solution, and the vial was sealed for reaction at 60 °C for 24 h. After cooling down to room temperature, the as-prepared electrode (denoted as NiFe-NDC//G) was then taken out, rinsed with copious DI-water and ethanol, and dried at 60 °C overnight.

Synthesis of Ni-NDC//G. The Ni-NDC//G electrode was prepared by following procedure to NiFe-NDC//G except that only 485 mg of $Ni(CH_3COO)_2·4H_2O$ was used as the metal precursor.

Synthesis of Fe-NDC//G. The Fe-NDC//G electrode was prepared by following procedure to NiFe-NDC//G except that only 788 mg of $Fe(NO_3)_3·9H_2O$ was used as the metal precursor.

Synthesis of NiFe-BDC//G. The synthesis of NiFe-BDC//G was similar with NiFe-BTC//G. Contrastingly, 250 mg of terephthalic acid and 120 mg of NaOH were dissolved in DI-water (75 mL) and rigorously stirred for 30 min to obtain the electrolyte to insert the organic ligands into the dilated graphite multilayers. The electrochemical intercalation time of organic ligands was set as 0.5 h. Subsequently, 715 mg of $NiCl_2·6H_2O$ and 1.21 g of $Fe(NO_3)_3·9H_2O$ were dissolved in a mixed solvent of DI-water and ethanol (100 mL, V:V=1:1) and stirred for 20 min to form transparent inorganic salt solution. The treated graphite was immersed into the above solution, and the vial was sealed for reaction at 60 ^{o}C for 24 h. After cooling down to room temperature, the as-prepared electrode (denoted as NiFe-BDC//G) was then taken out, rinsed with copious DI-water and ethanol.

Synthesis of Fe-BDC//G. The Fe-NDC//G electrode was prepared by following procedure to NiFe-NDC//G except that the electrochemical intercalation time of organic ligands was set as 1 h and only 1.21 g of $Fe(NO_3)_3·9H_2O$ was used as the metal precursor.

Synthesis of NiFe-CTC//G. The synthesis of NiFe-CTC//G was similar with NiFe-BTC//G. Contrastingly, 70 mg of 1,2,3,4-cyclobutanetetracarboxylic acid and 48 mg of NaOH were dissolved in DI-water (75 mL) and rigorously stirred for 30 min to obtain the electrolyte to insert the organic ligands into the dilated graphite multilayers. The electrochemical intercalation time of organic ligands was set as 2 h and the intercalation voltage was set as 8 V. Subsequently, 287 mg of $NiCl_2·6H_2O$ and 487 mg of $Fe(NO_3)_3·9H_2O$ were dissolved in a mixed solvent of DI-water and ethanol (100 mL, V:V=1:1) and stirred for 20 min to form transparent inorganic salt solution. The treated graphite was immersed into the above solution, and the vial was sealed for reaction at 60 ^{o}C for 24 h. After cooling down to room temperature, the as-prepared electrode (denoted as NiFe-CTC//G) was then taken out, rinsed with copious DI-water and ethanol, and dried at 60 ^{o}C overnight.

Synthesis of bulk NiFe-BTC. 420 mg of 1,3,5-benzenetricarboxylic acid and 240 mg of sodium hydroxide were dissolved in DI-water (75 mL) and vigorously stirred for 30 min to obtain transparent

organic solution and 713 mg of $NiCl_2 \cdot 6H_2O$ and 1.21 g of $Fe(NO_3)_3 \cdot 9H_2O$ were dissolved in a mixed solvent of DI-water and ethanol (100 mL, V:V=1:1) and stirred for 20 min to form transparent inorganic salt solution. The organic solution with 1,3,5-benzenetricarboxylic group molecules was stirred vigorously and the prepared inorganic Ni and Fe salt solution was added drop by drop. After the mixture of the two solutions, the suspension was further stirred for 15 min. Subsequently, the bulk NiFe-BTC powder was obtained through filtration and dried at 60 °C overnight. 10.0 mg of bulk NiFe-BTC powder was added to 1.0 mL of ethanol containing 10 μL Nafion aqueous solution (5 wt.%, Sigma-Aldrich), and dispersed by sonication for 30 min to generate a homogeneous ink. 100 μL of the catalyst ink was drop-casted on the surface of a carbon paper with a loading amount of 1.0 mg cm^{-2} and dried in air at room temperature.

Synthesis of bulk Ni-BTC. The bulk Ni-BTC powder was prepared by following procedure to bulk NiFe-BTC powder except that only 713 mg of $NiCl_2 \cdot 6H_2O$ was used as the metal precursor.

Synthesis of bulk Fe-BTC. The bulk Fe-BTC powder was prepared by following procedure to bulk NiFe-BTC powder except that only 1.21 g of $Fe(NO_3)_3 \cdot 9H_2O$ was used as the metal precursor.

Synthesis of bulk NiFe-NDC. 250 mg of 2,6-naphthalenedicarboxylic acid dipotassium was dissolved in DI-water (75 mL) and vigorously stirred for 30 min to obtain transparent organic solution. 485 mg of $Ni(CH_3COO)_2 \cdot 4H_2O$ and 788 mg of $Fe(NO_3)_3 \cdot 9H_2O$ were dissolved in a mixed solvent of DI-water and ethanol (100 mL, V:V=1:1) and stirred for 20 min to form transparent inorganic salt solution. The organic solution was stirred vigorously and the prepared inorganic Ni and Fe salt solution was added drop by drop. After the mixture of the two solutions, the suspension was further stirred for 15 min. Subsequently, the bulk NiFe-NDC powder was obtained through filtration, rinsed with copious DI-water and ethanol, and dried at 60 °C overnight.

Synthesis of bulk Ni-NDC. The bulk Ni-NDC powder was prepared by following procedure to bulk NiFe-NDC powder except that only 485 mg of $Ni(CH_3COO)_2 \cdot 4H_2O$ was used as the metal precursor.

Synthesis of bulk Fe-NDC. The bulk Fe-NDC powder was prepared by following procedure to bulk NiFe-NDC powder except that only 788 mg of $Fe(NO_3)_3 \cdot 9H_2O$ was used as the metal precursor.

Synthesis of bulk NiFe-BDC. 250 mg of terephthalic acid and 120 mg of NaOH were dissolved in DI-water (75 mL) and vigorously stirred for 30 min to obtain transparent organic solution. 715 mg of $NiCl_2 \cdot 6H_2O$ and 1.21 g of $Fe(NO_3)_3 \cdot 9H_2O$ were dissolved in a mixed solvent of DI-water and ethanol (100 mL, V:V=1:1) and stirred for 20 min to form transparent inorganic salt solution. The organic solution was stirred vigorously and the prepared inorganic Ni and Fe salt solution was added drop by drop. After the mixture of the two solutions, the suspension was further stirred for 15 min. Subsequently, the bulk NiFe-BDC powder was obtained through filtration, rinsed with copious DI-water and ethanol, and dried at 60 ^{o}C overnight.

Synthesis of bulk Fe-BDC. The bulk Fe-BDC powder was prepared by following procedure to bulk NiFe-BDC powder except that only 1.21 g of $Fe(NO_3)_3 \cdot 9H_2O$ was used as the metal precursor.

Synthesis of bulk NiFe-CTC. 70 mg of 1,2,3,4-cyclobutanetetracarboxylic acid and 48 mg of NaOH were dissolved in DI-water (75 mL) and vigorously stirred for 30 min to obtain transparent organic solution. 287 mg of $NiCl_2 \cdot 6H_2O$ and 487 mg of $Fe(NO_3)_3 \cdot 9H_2O$ were dissolved in a mixed solvent of DI-water and ethanol (100 mL, V:V=1:1) and stirred for 20 min to form transparent inorganic salt solution. The organic solution was stirred vigorously and the prepared inorganic Ni and Fe salt solution was added drop by drop. After the mixture of the two solutions, the suspension was further stirred for 15 min. Subsequently, the bulk NiFe-CTC powder was obtained through filtration, rinsed with copious DI-water and ethanol, and dried at 60 ^{o}C overnight.

Synthesis of Ir/C//G. The commercial graphite foil was treated and electrochemically exfoliated by following procedure to NiFe-BTC//G-2h. 10.0 mg of Ir/C was added to 1.0 mL of ethanol containing 10 μL Nafion aqueous solution (5 wt.%, Sigma-Aldrich), and dispersed by sonication for 30 min to generate a homogeneous ink. 100 μL of the catalyst ink was drop-casted on the surface of the expanded graphite foil with a loading amount of 1.0 mg cm^{-2} and dried in air at room temperature.

Synthesis of RuO_2//G. The RuO_2//G electrode was prepared by following procedure to Ir/C//G except that 10.0 mg of RuO_2 was added instead of Ir/C.

The EXAFS signals fitting and wavelet transform (WT) analysis

The obtained XAFS data was processed in Athena (version 0.9.26) for background, pre-edge line and post-edge line calibrations. Then Fourier transformed fitting was carried out in Artemis (version 0.9.26) [1212121212].[1,2] The k^3 weighting, k-range of ~3.0-10.0 $Å^{-1}$ and R range of ~1.0-2.4 Å were used for the fitting. The models of Fe foil, Fe_2O_3 and NiFe-BTC//G were used to calculate the simulated scattering paths. The four parameters, coordination number, bond length, Debye-Waller factor and E0 shift (CN, R, σ^2, ΔE_0) were fitted and partially fixed. For Wavelet Transform analysis, the $\chi(k)$ exported from Athena was imported into the Hama Fortran code.[3] The parameters were listed as follow: R range, 0-3 Å, k range, 3-13.9 $Å^{-1}$; k weight, 3; and Morlet function with $\kappa=5$, $\sigma=1$ was used as the mother wavelet to provide the overall distribution.

Free energy corrections

Free energy corrections were applied following the equation below, including the effect from zero-point energy, pressure, inner energy, and entropy:

$$E_{cor} = E_{cor}^{ZPE} + E_{cor}^{U} + E_{cor}^{P} + E_{cor}^{S} \tag{1}$$

where E_{cor}^{ZPE} refers to the correction of zero-point energy, E_{cor}^{U} and E_{cor}^{P} refer to the correction of temperature (inner energy correction) and pressure and E_{cor}^{S} refers to the correction of entropy. For adsorbates, only vibrational motion was considered, resulting in that the free energy corrections were only depended on the vibrational frequency and temperature, while, all translational, rotational and vibrational motions were considered for gas molecules.[4] The free energy corrections were carried out at 298 K with the pressure of H_2 and H_2O to be 1.0 and 0.035 bar respectively.

The free energy of $O_2(g)$ was calculated based on the free energy of $H_2(g)$ and $H_2O(l)$, following:

$$G_{O_2(g)} = 1.23\ \text{eV} \times 4 + 2G_{H_2O(l)} - 2G_{H_2(g)} \tag{2}$$

The chemical potential of OH^- was determined as:

$$G_{OH^-} = G_{H_2O(l)} - G_{H^+} \tag{3}$$

In equilibrium, the chemical potential of a pair of ($H^+ + e^-$) at 0 V vs. RHE was referred to ½ chemical potential of H_2 molecule in light of the computational hydrogen electrode (CHE) approximation.[5]

Solvent effect corrections

The solvation effect was corrected for all the adsorbates due to the formation of H-bonds in solvent, where a stabilization on adsorptions was reported.[6,7] In this work, the solvent effects were carried out based on an implicit model, namely VASPsol[8,9], where a stabilization for O*, OH* and OOH* was estimated to be 0.12, 0.14 and 0.42 eV, respectively. Note that, it is also worth to pay close attention on the solvent effect in different kinds of system (i.e. open system and confined system). A rigorous investigation is recommended to double check the energy effect from the solvent effect, when different kind of catalytic system were studied (Supplementary Fig. 41 and Supplementary Table 11).

Reaction free energy calculations

The four electron-proton transfer steps were considered (Supplementary Fig. 42) as elementary steps for OER in alkaline condition, following:

$$OH^- + * \rightarrow OH^* + e^- \quad (4)$$

$$OH^* + OH^- \rightarrow O^* + H_2O(l) + e^- \quad (5)$$

$$O^* + OH^- \rightarrow OOH^* + e^- \quad (6)$$

$$OOH^* + OH^- \rightarrow * + O_2(g) + H_2O(l) + e^- \quad (7)$$

The adsorption free energies (G_{O^*}, G_{OH^*} and G_{OOH^*}) of three adsorbates were calculated, and the reaction free energies (ΔG_1 - ΔG_4 refer to elementary steps of Supplementary Equations 4-7) at 0 V vs RHE can be calculated:

$$\Delta G_1 = G_{OH^*} \quad (8)$$

$$\Delta G_2 = G_{O^*} - G_{OH^*} \quad (9)$$

$$\Delta G_3 = G_{OOH^*} - G_{O^*} \quad (10)$$

$$\Delta G_4 = 4.92 - G_{OOH^*} \quad (11)$$

According to the effect from the electrode potential, the reaction free energy of electrochemal steps was highly affected, where the potential dependent reaction free energy can be calculated based on the CHE approximation:[2]

$$\Delta G_U = \Delta G_{U_0} + e\,(U - U_0) \quad (12)$$

where U and U_0 refer to the considered electrode potential U and 0 V vs RHE, respectively. The theoretical activity was evaluated based on the limiting energy in the RPD (G_{RPD}-limiting), which is defined as the highest reaction free energy within the OER pathway (see the reaction free energy calculations in method):

$$G_{RPD}\text{-limiting} = \max\,(\Delta G_1, \Delta G_2, \Delta G_3, \Delta G_4) \quad (13)$$

Supplementary Figures

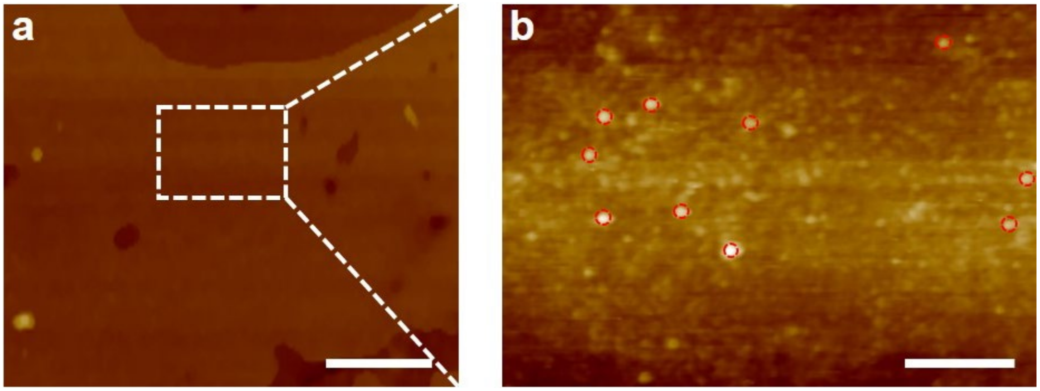

Supplementary Fig. 1 | AFM images for NiFe-BTC//G-2h. AFM image of (**a**) NiFe-BTC//G-2h electrode, and (**b**) enlarged AFM image of the white rectangle in (**a**). Scale bars, 1.0 µm (**a**), 200 nm (**b**). Red circles: Nanoparticles of NiFe-BTC.

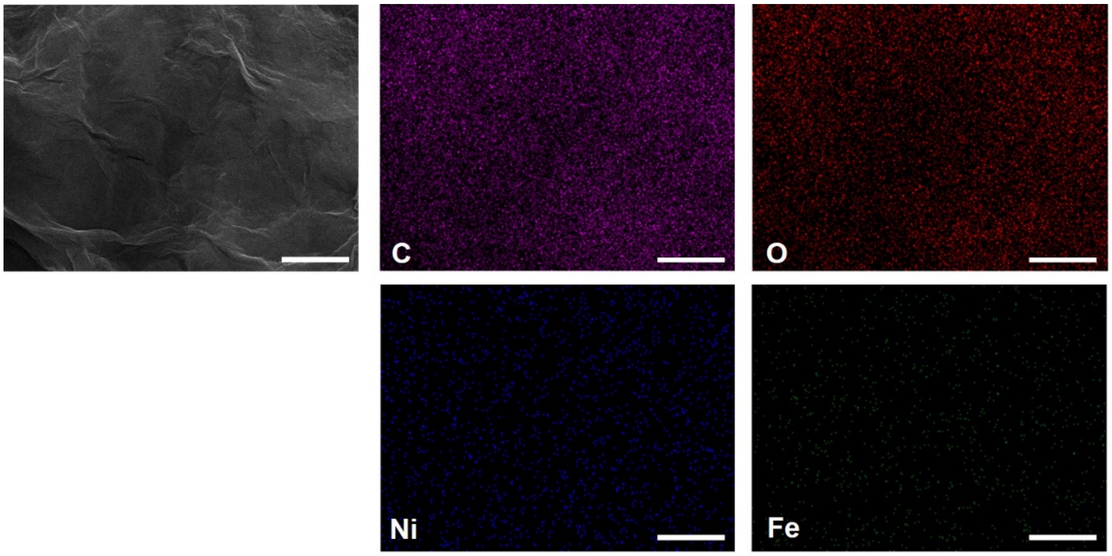

Supplementary Fig. 2 | SEM and element mapping images of NiFe-BTC//G-2h electrode. Scale bars, 2.0 μm.

The scanning electron microscopy (SEM) and energy dispersive X-ray spectroscopy (EDX) element mapping images demonstrated the uniform dispersion of C, O, Ni, and Fe elements throughout the entire inlayer of NiFe-BTC//G, which indicates that the NiFe-BTC had been intercalated into the space of graphite foil.

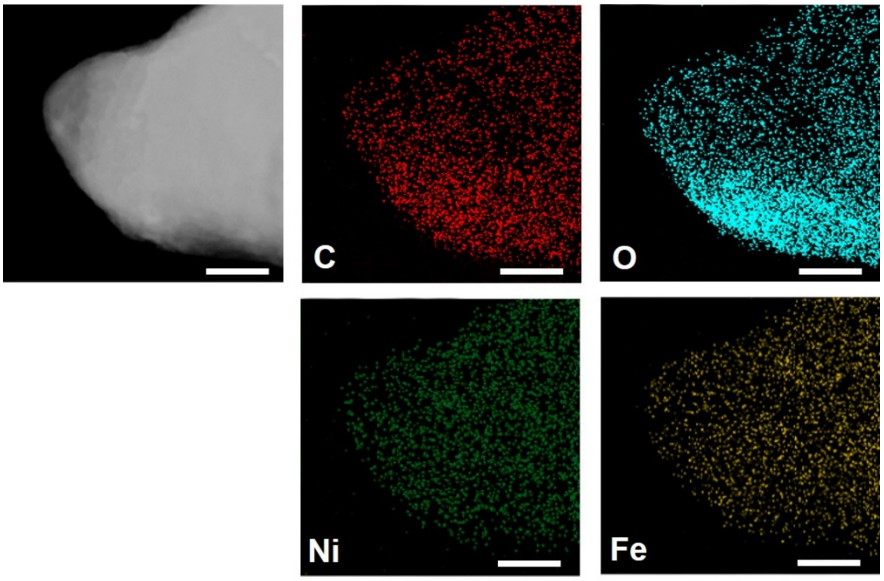

Supplementary Fig. 3 | HAADF-STEM image and EDX element mapping images of NiFe-BTC//G-2h. Scale bars, 500 nm. The high-angle annular dark-field scanning TEM (HAADF STEM) image and EDX element mapping images show that the densities of C, O, Fe, and Ni are uniformly distributed throughout the entire NiFe-BTC//G-2h electrode, proving the presence of physical NiFe-BTC.

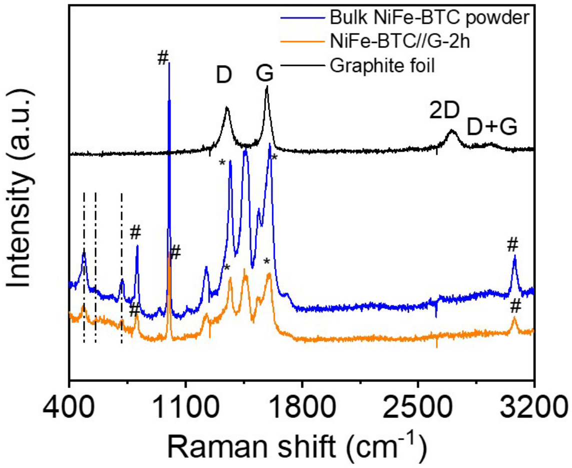

Supplementary Fig. 4 | Raman spectra of bulk NiFe-BTC powder, NiFe-BTC//G-2h, and graphite foil. (* corresponds to peaks of graphite layers; # corresponds to peaks of 1,3,5-benzenetricarboxylic group). The Raman spectra in Supplementary Fig. 4 disclose the existence of invariant and primary peaks for bulk NiFe-BTC powder. Comparatively, the Raman peak intensities of NiFe-BTC//G-2h significantly reduce, indicating the intercalation of NiFe-BTC into graphite multilayers during electrochemical intercalation,[10] which could be also proved by the low intensity of graphene D band (1368 cm^{-1}) over G band (1604 cm^{-1}).[10-12] Further, three Raman peaks centered at 485, 557 and 717 cm^{-1} of both bulk NiFe-BTC powder and NiFe-BTC//G-2h could be correlated with the characteristic bands of M-O or M-O-M (M represents Ni^{2+} or Fe^{3+}).[13,14] The peaks at around 1760-1670 cm^{-1} could be assigned to the coordinations of Ni or Fe ions with 1,3,5-benzenetricarboxylate ligands.[15]

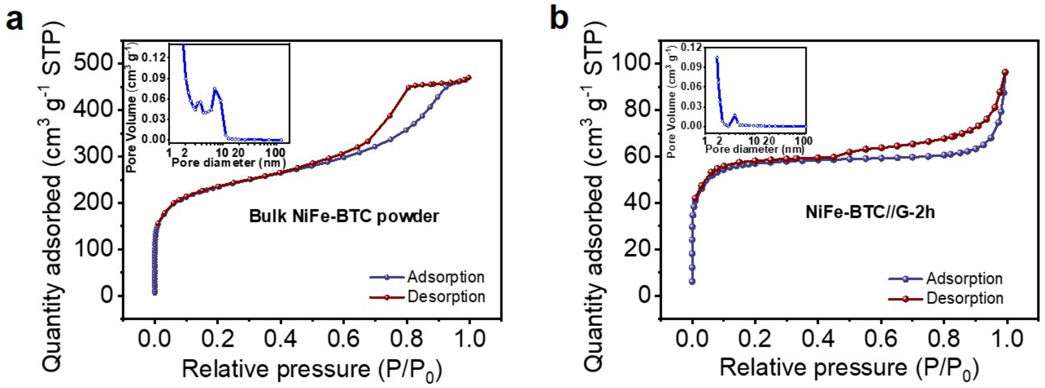

Supplementary Fig. 5 | The N_2 adsorption and desorption isotherm curves. The N_2 adsorption and desorption isotherm curves of (**a**) bulk NiFe-BTC powder and (**b**) NiFe-BTC//G-2h. Inset: corresponding pore size distributions of bulk NiFe-BTC powder and NiFe-BTC//G-2h. The BET results of bulk NiFe-BTC powder and NiFe-BTC//G-2h are analyzed from their N_2 adsorption-desorption curves. Supplementary Fig. 4 indicates that both bulk NiFe-BTC powder and NiFe-BTC//G-2h show type-IV isotherms, while NiFe-BTC//G-2h possesses part of partial micropores and mostly mesopores or macropores.[16,17] The NiFe-BTC//G-2h exhibits a BET specific surface area of 762.7 m^2 g^{-1} with a total pore volume of 0.15 cm^3 g^{-1}.

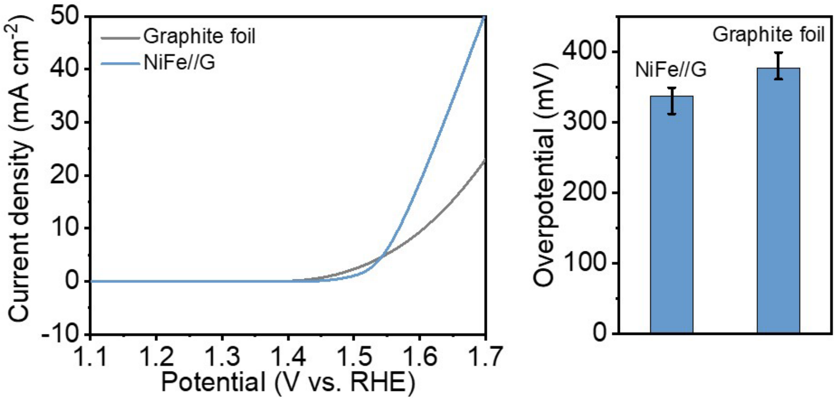

Supplementary Fig. 6 | OER activities of NiFe//G and graphite foil. (**a**) LSV plots obtained with NiFe//G and graphite foil for OER at 10 mV s^{-1} in 1.0 M KOH. (**b**) Comparison of the required voltages at 10 mA cm^{-2} for NiFe//G and graphite foil.

The expanded graphite foil (denoted as graphite foil) is directly immersed in Ni and Fe salt solution without the introduction of organic ligands (denoted as NiFe//G) to verify the possibilities of carboxyl groups (-COOH) from graphene to coordinate with metal ions. Both the graphite foil and NiFe//G were tested for 20 times to obtain the margin of error. As shown in Supplementary Fig. 6, the NiFe//G has an averagely lower overpotential of 338 mV to achieve the current density of 10 mA cm^{-2} compared to the graphite foil (376 mV at 10 mA cm^{-2}). However, their overpotentials are both much higher than that of NiFe-BTC//G (106 mV at 10 mA cm^{-2}), which proved the restricted contribution of the coordinate between the carboxyl groups (-COOH) formed in graphene with metal ions.

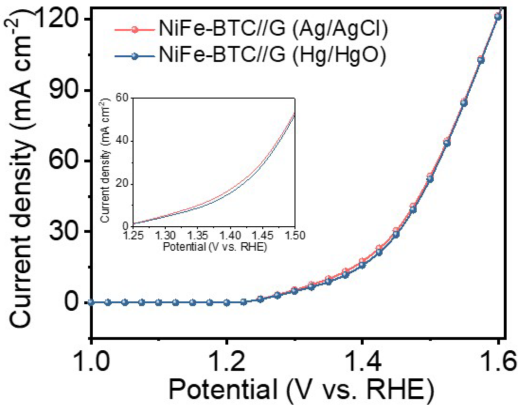

Supplementary Fig. 7 | OER polarization curves of NiFe-BTC//G with different reference electrodes. LSV plots obtained with NiFe-BTC//G for OER at 10 mV s^{-1} in 1.0 M KOH. Red: Ag/AgCl (saturated KCl) as the reference electrode; Blue: Hg/HgO as the reference electrode. Inset: A close-up of LSV plots.

As shown in Supplementary Fig. 7, the NiFe-BTC//G displayed similar overpotentials when the Ag/AgCl (~118 mV) and Hg/HgO (~130 mV) were worked as reference electrodes, respectively. The Ag/AgCl (saturated KCl) electrode was proved to be stable in alkaline solution when protected by the salt bridge, which can be attributed to the directly contact of Ag/AgCl with the saturated KCl solution rather than the alkaline solution. The chronopotentiometry measurement for the NiFe-BTC//G displayed an insignificant potential change over 150 h of continuous reaction at a constant current density of 10 mA cm^{-2} in 1.0 M KOH when employing Ag/AgCl (saturated KCl) electrode as reference (Fig. 2d), which further confirmed the relative stability of Ag/AgCl (saturated KCl) electrode in alkaline solution.

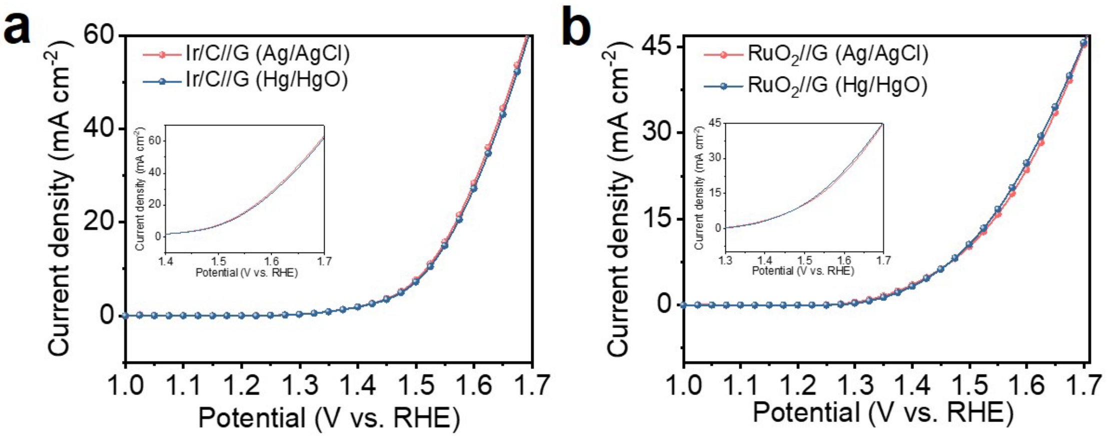

Supplementary Fig. 8 | OER polarization curves of Ir/C//G and RuO_2//G with different reference electrodes. LSV plots obtained with (**a**) Ir/C//G and (**b**) RuO_2//G for OER at 10 mV s^{-1} in 1.0 M KOH. Red: Ag/AgCl (saturated KCl) as the reference electrode; Blue: Hg/HgO as the reference electrode. Inset: Close-ups of LSV plots.

As shown in Supplementary Fig. 8a, the Ir/C//G displayed similar overpotentials when the Ag/AgCl (~287 mV) and Hg/HgO (~292 mV) were worked as reference electrodes, respectively. Also, when employing the Ag/AgCl (saturated KCl) and Hg/HgO electrodes as references, the RuO_2//G showed comparable overpotentials of 267 and 264 mV, respectively (Supplementary Fig. 8b). The OER performances of noble metal based electrocatalysts further proved the stability of Ag/AgCl (saturated KCl) electrode in 1.0 M KOH.

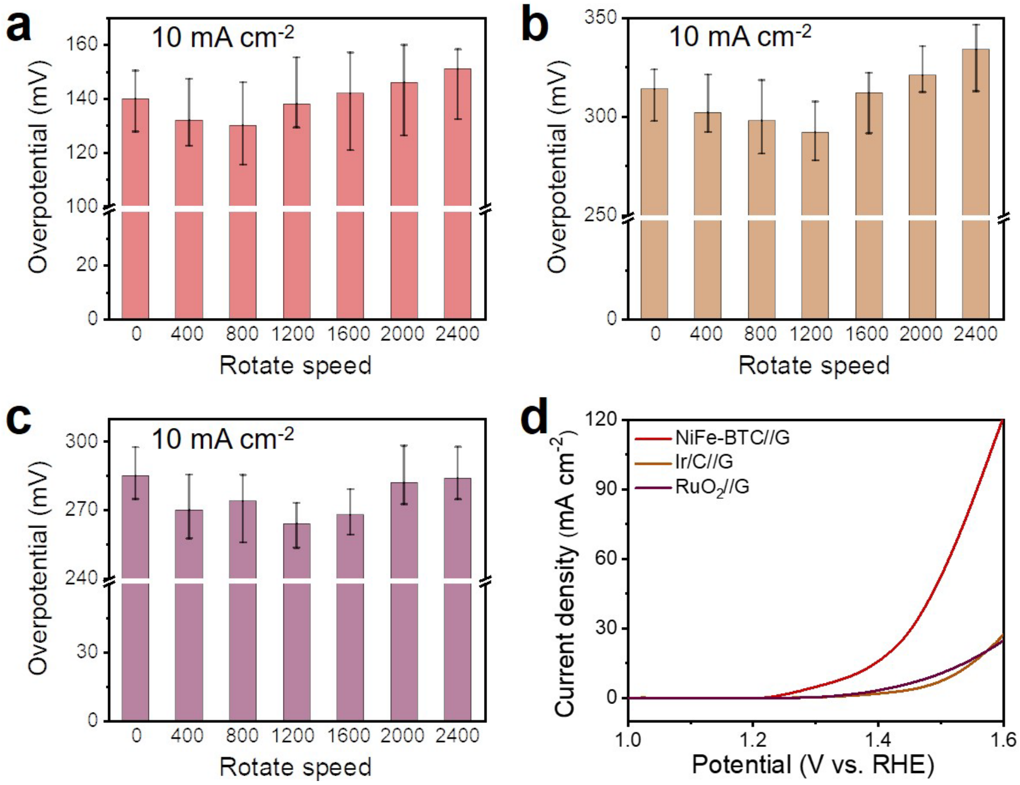

Supplementary Fig. 9 | Overpotential comparisons of NiFe-BTC//G, Ir/C//G, and RuO_2//G at different rotate speed. Overpotentials of (**a**) NiFe-BTC//G, (**b**) Ir/C//G, and (**c**) RuO_2//G tested under rotation at the rotate speed from 0 to 2400 rpm for OER at 10 mV s^{-1} in 1.0 M KOH using Hg/HgO as the reference electrode. (**d**) LSV plots obtained with NiFe-BTC//G, Ir/C//G, and RuO_2//G under rotation for OER at 10 mV s^{-1} in 1.0 M KOH using Hg/HgO as the reference electrode.

As shown in Supplementary Fig. 9a-c, the overpotentials of NiFe-BTC//G, Ir/C//G, and RuO_2//G relocate with the rotate speed, which are within ranges of 130-151 mV, 292-334 mV, and 264-285 mV respectively. The NiFe-BTC//G displays the smallest overpotential of ~130 mV at 10 mA cm^{-2} at the rotate speed of 800 rpm. While the Ir/C//G and RuO_2//G display the overpotential of ~292 and ~264 mV at the rotate speed of 1200 rpm, respectively (Supplementary Fig. 9d). The OER performances of the NiFe-BTC//G catalyst at low rotate speed are restricted which could be attributed to the block of metal active sites by a large amount of bubbles. Accordingly, high rotate speed may restrict the access of water molecule into the confined space of electrodes and reduce the contact time of intermediates with metal active sites, which weakens the OER kinetics.

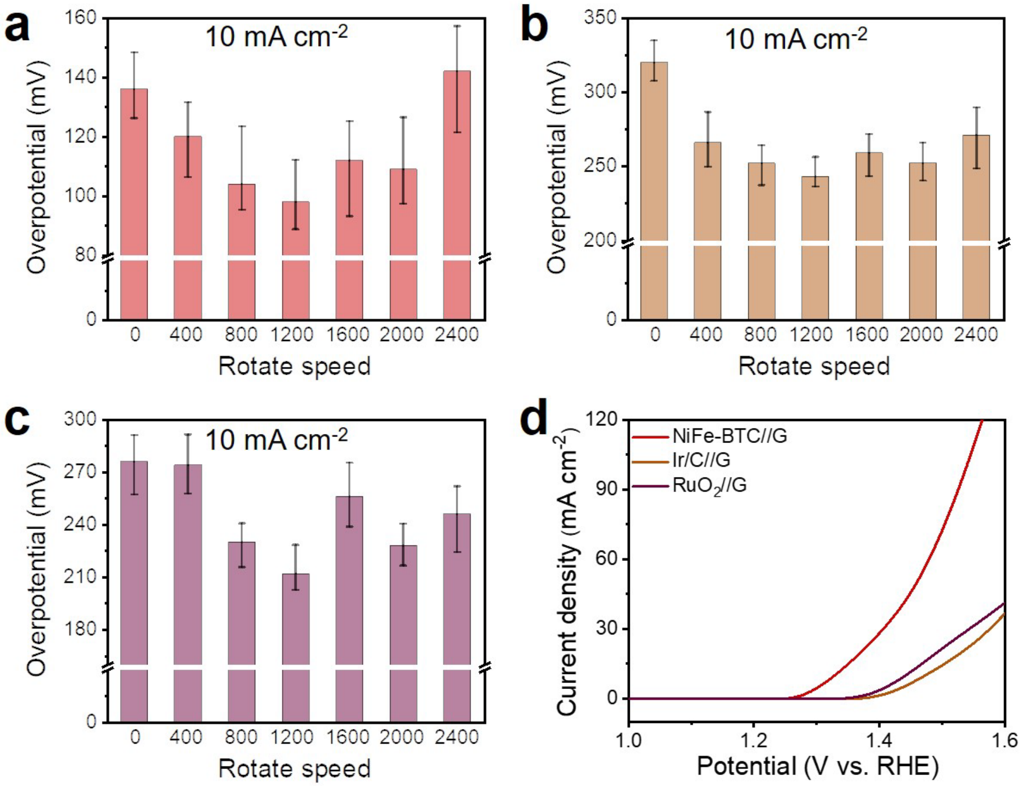

Supplementary Fig. 10 | Overpotential comparisons of NiFe-BTC//G, Ir/C//G, and RuO_2//G tested through an RDE technique at different rotate speed. Overpotentials of (**a**) NiFe-BTC//G, (**b**) Ir/C//G, and (**c**) RuO_2//G tested through an RDE technique under rotation at the rotate speed from 0 to 2400 rpm for OER at 10 mV s^{-1} in 1.0 M KOH using Hg/HgO as the reference electrode. (**d**) LSV plots obtained with NiFe-BTC//G, Ir/C//G, and RuO_2//G tested through the RDE technique for OER at 10 mV s^{-1} in 1.0 M KOH using Hg/HgO as the reference electrode.

As shown in Supplementary Fig. 10a-c, the overpotentials of NiFe-BTC//G, Ir/C//G, and RuO_2//G were investigated within ranges of 98-142 mV, 243-320 mV, and 212-276 mV respectively. The NiFe-BTC//G shows the smallest overpotential of ~98 mV at 10 mA cm^{-2} through the RDE technique at 1200 rpm. While the Ir/C//G and RuO_2//G show the overpotential of ~243 and ~212 mV through the RDE technique at 1200 rpm, respectively (Supplementary Fig. 10d). The high overpotential obtained at low rotate speed could be attributed to the generated bubbles that block the active sites. While the excessive rotate speed with large shear force would introduce undesired vortex which impedes the contact of intermediates with metal active sites.

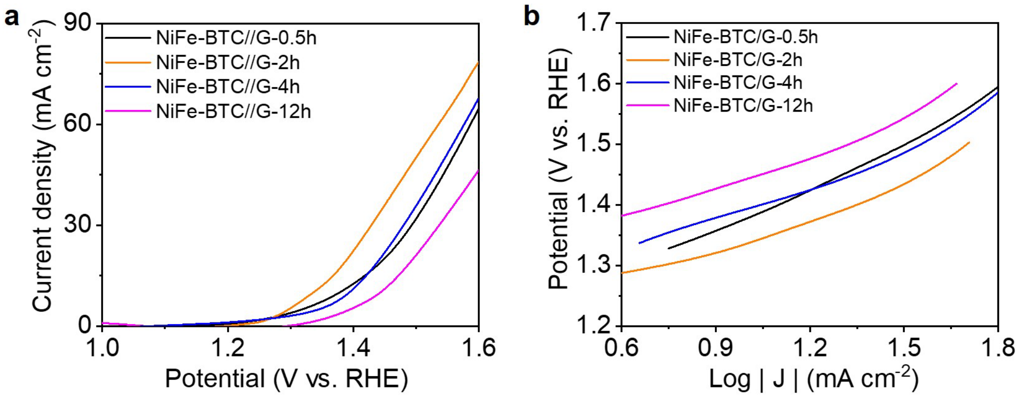

Supplementary Fig. 11 | OER activities of NiFe-BTC//G-X. (**a**) LSV plots obtained with NiFe-BTC//G-*X* for OER at 10 mV s^{-1} in 1.0 M KOH, *X* = 0.5 h, 2 h, 4 h, and 12 h. (**b**) Tafel plots obtained with NiFe-BTC//G-*X*, *X* = 0.5 h, 2 h, 4 h, and 12 h. A series of NiFe-BTC//G-*X* electrodes with different incremental electrochemical intercalation time of organic ligands (0.5 to 4 h) were directly employed to trigger the anodic reaction of water splitting without carbonization treatment. The overpotentials of NiFe-BTC//G-*X* from 0.5 h to 12 h at 10 mA cm^{-2} in 1.0 M KOH solution were confirmed as 152 mV, 106 mV, 166 mV, and 212 mV, respectively (Supplementary Fig. 5A). The corresponding Tafel slopes were calculated as 58 mV dec^{-1}, 40 mV dec^{-1}, 56 mV dec^{-1}, and 48 mV dec^{-1}, respectively (Supplementary Fig. 11b). The prepared NiFe-BTC//G-2h with electrochemical intercalation time of 2 h was proved to display best electrocatalytic performances to conduct OER.

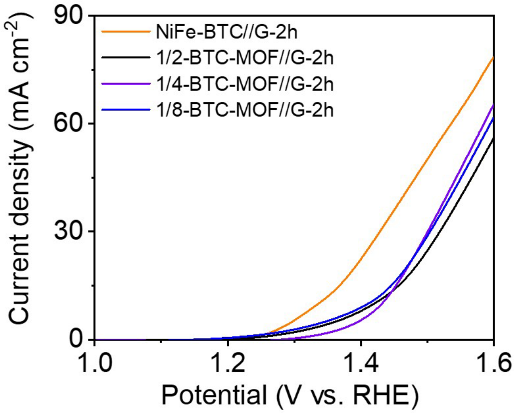

Supplementary Fig. 12 | OER polarization curves of NiFe-BTC//G-2h with different Ni/Fe ratios. LSV plots obtained with Ni-BTC//G-2h, 1/2-Ni-BTC//G-2h, 1/4-Ni-BTC//G-2h, and 1/8-Ni-BTC//G-2h for OER at 10 mV s^{-1} in 1.0 M KOH. A series of NiFe-BTC//G-2h electrodes were prepared through immersing in mixed solution of metallic salts (nickel chloride and iron nitrate) with different Ni/Fe ratios of 1/1, 1/2, 1/4, and 1/8 (denoted as Ni-BTC//G-2h, 1/2-Ni-BTC//G-2h, 1/4-Ni-BTC//G-2h, and 1/8-Ni-BTC//G-2h). The overpotentials of Ni-BTC//G-2h, 1/2-Ni-BTC//G-2h, 1/4-Ni-BTC//G-2h, and 1/8-Ni-BTC//G-2h at 10 mA cm^{-2} in 1.0 M KOH solution were confirmed as 106 mV, 190 mV, 202 mV, and 181 mV, respectively. The prepared NiFe-BTC//G-2h with Ni/Fe ratio of 1/1 was proved to display best electrocatalytic performances to conduct OER.

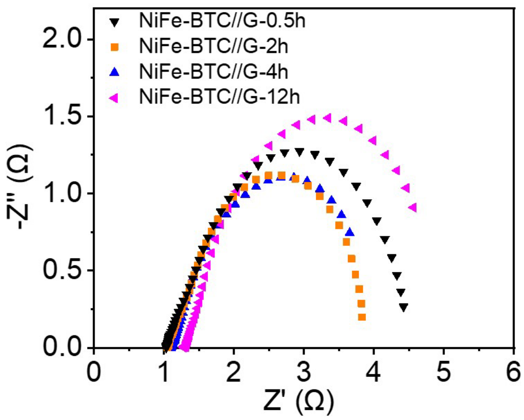

Supplementary Fig. 13 | EIS Nyquist plots of NiFe-BTC//G-*X*, *X* = 0.5 h, 2 h, 4 h, and 12 h. *X* represents different electrochemical intercalation time of organic ligands (0.5 to 12 h). The rapid electron transfer abilities for a series of NiFe-BTC//G-*X* electrodes are promoted by EIS, where NiFe-BTC//G-2h displays the smallest charge transfer resistance (R_{ct}) value of 0.46 Ω compared to NiFe-BTC//G-0.5h (1.23 Ω), NiFe-BTC//G-4h (0.91 Ω), and NiFe-BTC//G-12h (1.36 Ω) (Supplementary Table 4).

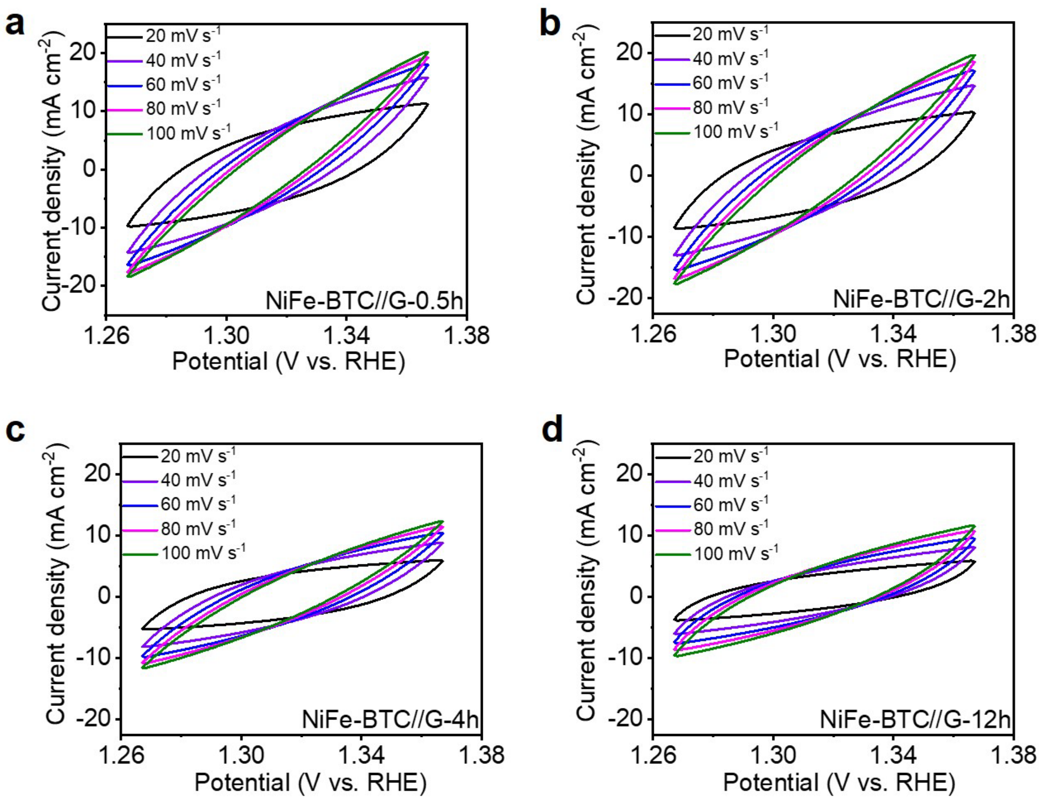

Supplementary Fig. 14 | CV curves of NiFe-BTC//G-*X*, *X* = 0.5 h, 2 h, 4 h, and 12 h. *X* represents different electrochemical intercalation time of organic ligands (0.5 to 12 h).CV curves of (**a**) NiFe-BTC//G-0.5h, (**b**) NiFe-BTC//G-2h, (**c**) NiFe-BTC//G-4h, and (**d**) NiFe-BTC//G-12h at different scan rates in 1.0 M KOH solution.

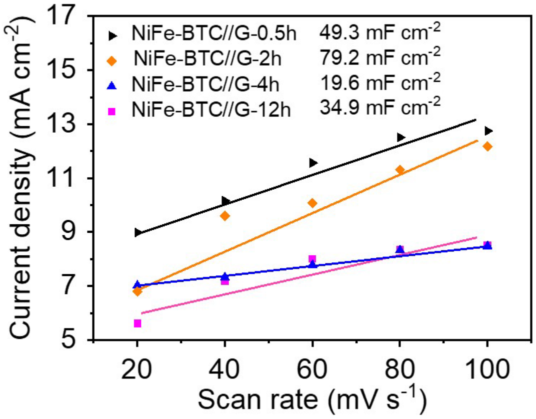

Supplementary Fig. 15 | ECSAs of NiFe-BTC//G-*X*, *X* = 0.5 h, 2 h, 4 h, and 12 h. *X* represents different electrochemical intercalation time of organic ligands (0.5 to 12 h). Supplementary Fig. 15 shows that the highest C_{dl} value of 79.2 mF cm^{-2} was observed for NiFe-BTC//G-2h as compared to those of NiFe-BTC//G-0.5h (49.3 mF cm^{-2}), NiFe-BTC//G-4h (19.6 mF cm^{-2}), and NiFe-BTC//G-12h (34.9 mF cm^{-2}), revealing that the NiFe-BTC//G-2h electrode possesses highly exposed active sites.

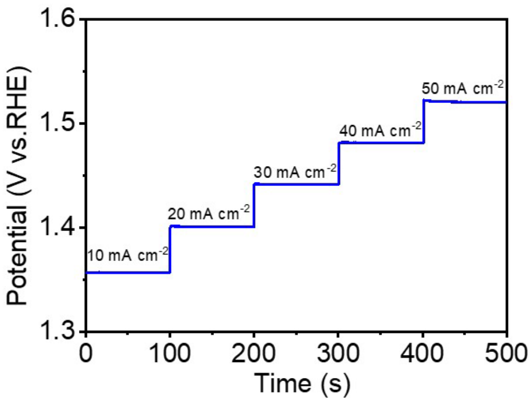

Supplementary Fig. 16 | Multi-step chronopotentiometric curve of NiFe-BTC//G-2h. The multi-step chronopotentiometric curve for NiFe-BTC//G-2h reveals that the corresponding potential is steady and responds quickly at each step from 10 to 50 mA cm^{-2}, suggesting an outstanding mass transport property and mechanical robustness.

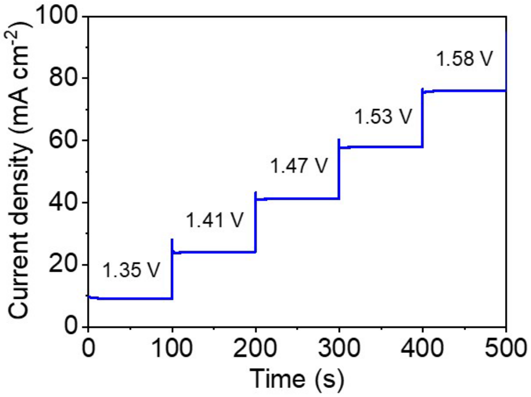

Supplementary Fig. 17 | Multi-step chronoamperometric curve of NiFe-BTC//G-2h. The multi-step chronoamperometric curve for NiFe-BTC//G-2h reveals that the corresponding current is steady and responds quickly at each step from 1.35 to 1.58 V, suggesting an outstanding mass transport property and mechanical robustness.

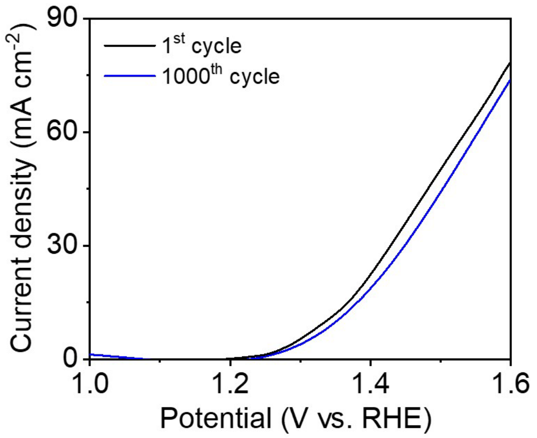

Supplementary Fig. 18 | Polarization curves of NiFe-BTC//G-2h before and after 1000 CV cycles. The polarization curve of NiFe-BTC//G-2h demonstrates an inconspicuous loss even after 1000 CV cycles, implying its high stability and durability for OER in alkaline media.

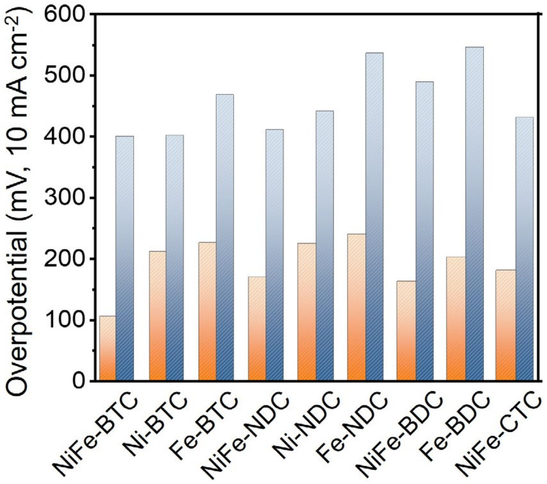

Supplementary Fig. 19 | Overpotential comparisons of various MOFs containing carboxylic esters synthesized by our strategy with their bulk phases. Red column: overpotentials of MOFs prepared through our synthesis strategy; Green column: overpotentials of bulk MOFs. In electrochemical test, we found various graphene multilayers confined MOFs (i.e., MOF//G) always gave much lower overpotentials in comparison of their bulk MOF powders. Particularly, the NiFe-BTC//G displays a record low overpotential of 106 mV at 10 mA cm^{-2} in alkaline solution.[18-21] The corresponding OER performances are summarized in Supplementary Table 5.

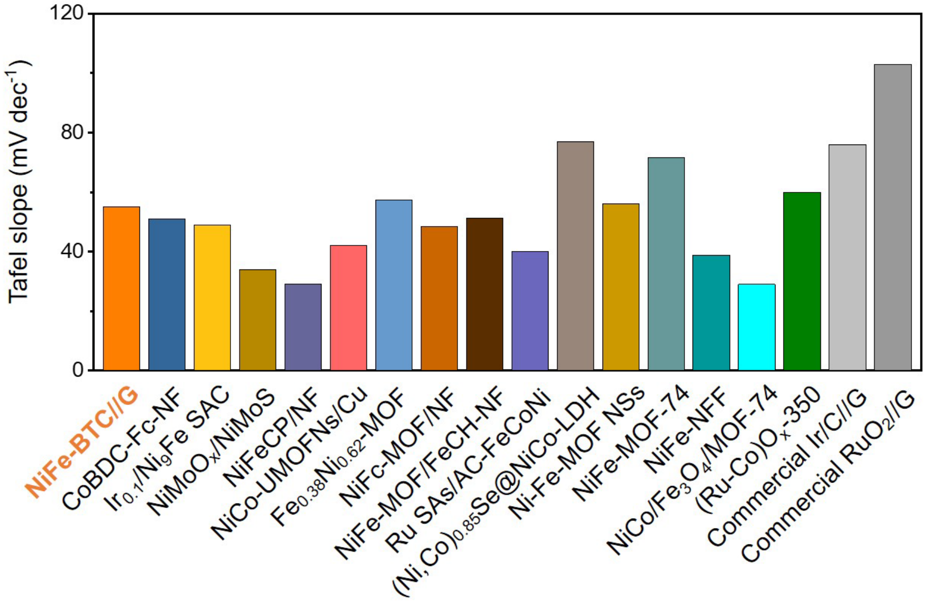

Supplementary Fig. 20 | Tafel slope comparisons of NiFe-BTC//G-2h with other state-of-the-art OER electrocatalysts. The NiFe-BTC//G-2h shows much lower Tafel slope of 55 mV dec^{-1} compared with the pristine NiFe-BTC (189 mV dec^{-1}), which is comparable with other state-of-the-art electrocatalysts. It indicates that our strategy of confining MOFs into graphite multilayers could provide more favorable reaction kinetics toward water oxidation reaction. The corresponding data are summarized in Supplementary Table 7.

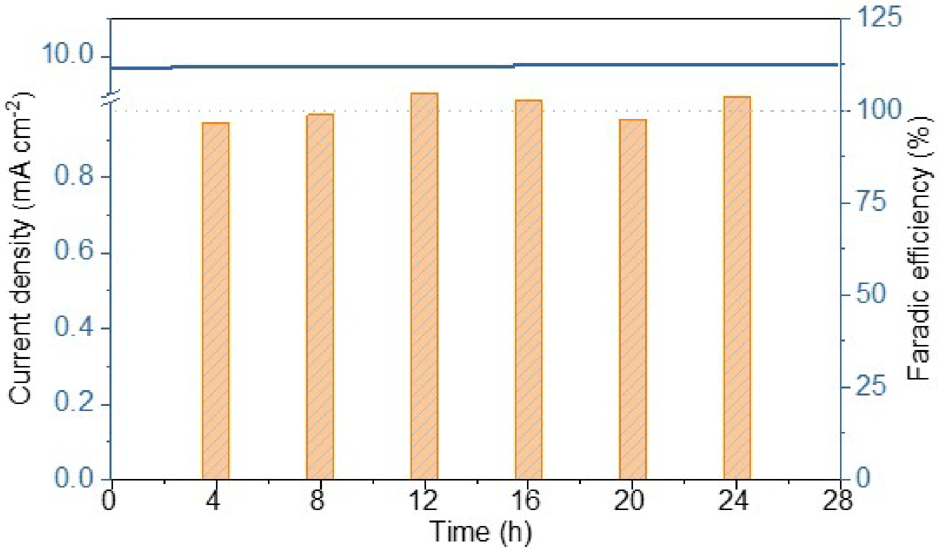

Supplementary Fig. 21 | Faradic efficiencies. Faradic efficiencies at overpotential of 106 mV for NiFe-BTC//G-2h and related current density in 24 h.

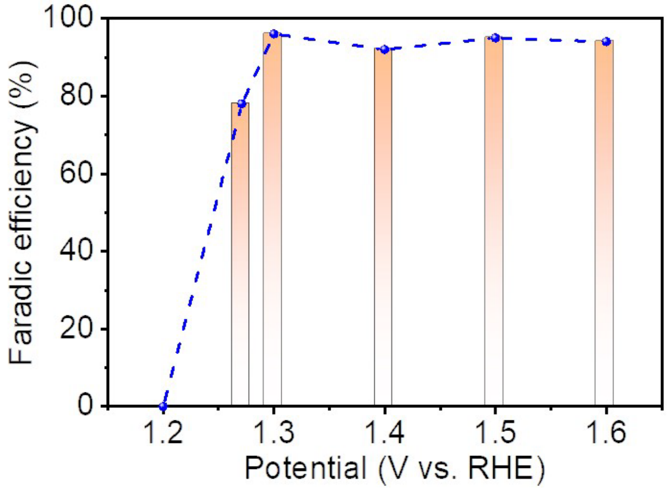

Supplementary Fig. 22 | Faraday efficiencies for NiFe-BTC//G tested from 1.2 to 1.6 V vs. RHE.

The O_2 Faraday efficiency tested at potential of 1.271 V vs. RHE is ~78%, which indicates the low onset potential of NiFe-BTC//G. Moreover, the O_2 Faraday efficiencies for NiFe-BTC//G from 1.3 to 1.6 V maintain 94 ± 2%, proving the efficient O_2 production during electrocatalysis.

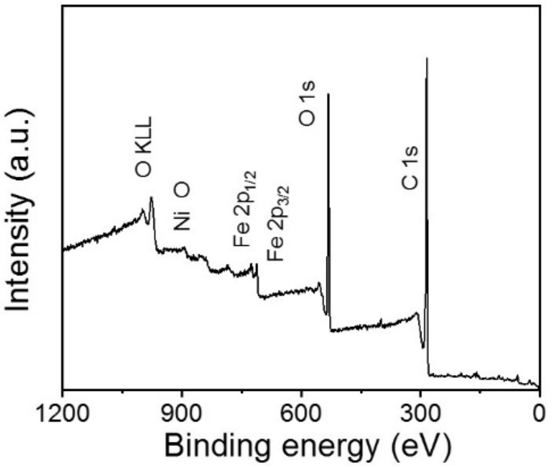

Supplementary Fig. 23 | XPS survey spectrum of NiFe-BTC//G-2h. The XPS survey spectrum of NiFe-BTC//G indicates the co-existence of C, O, Ni and Fe elements.

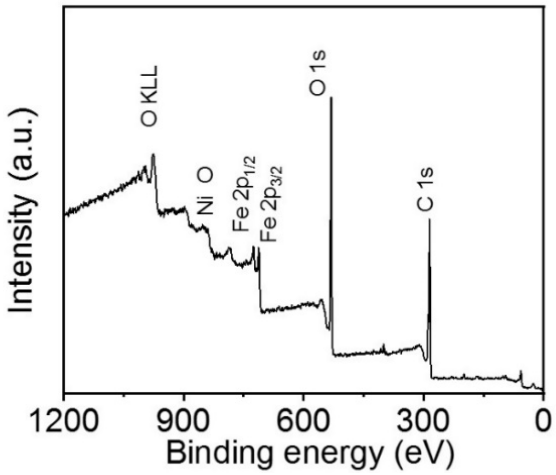

Supplementary Fig. 24 | XPS survey spectrum of bulk NiFe-BTC powder. The XPS survey spectrum of bulk NiFe-BTC indicates the co-existence of C, O, Ni and Fe elements.

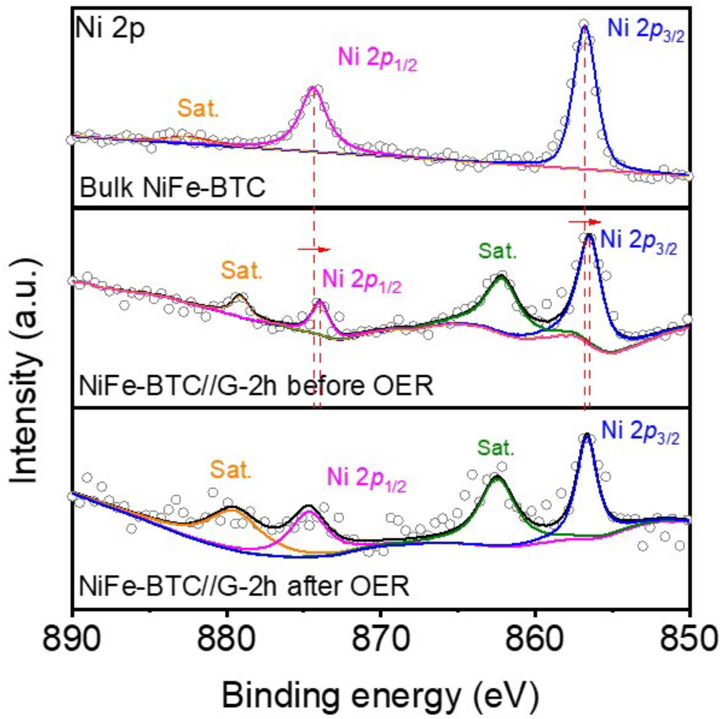

Supplementary Fig. 25 | The high-resolution XPS signals of Ni 2*p*. The high-resolution XPS signals of Ni 2*p* for bulk NiFe-BTC, NiFe-BTC//G-2h before OER, and NiFe-BTC//G-2h after OER.

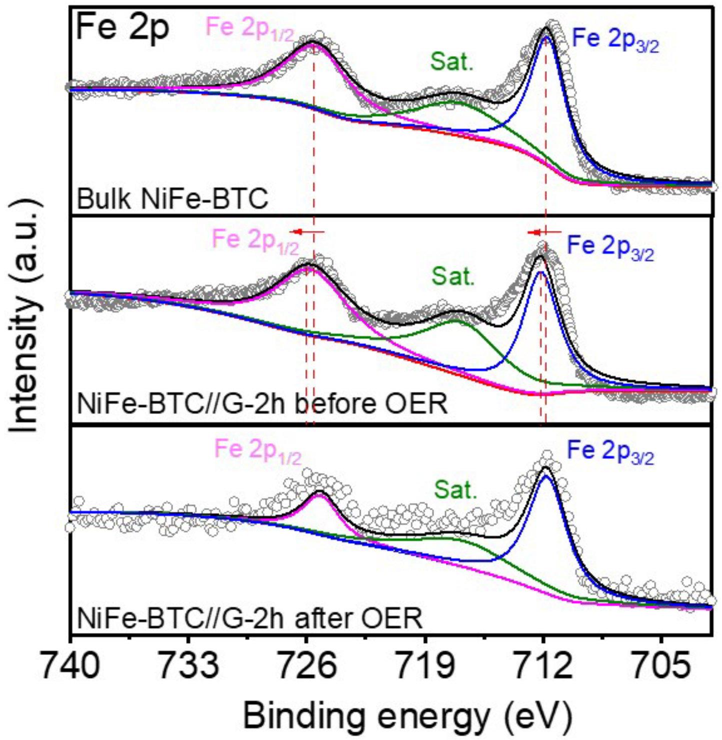

Supplementary Fig. 26 | The high-resolution XPS signals of Fe 2*p*. The high-resolution XPS signals of Fe 2*p* for bulk NiFe-BTC, NiFe-BTC//G-2h before OER, and NiFe-BTC//G-2h after OER.

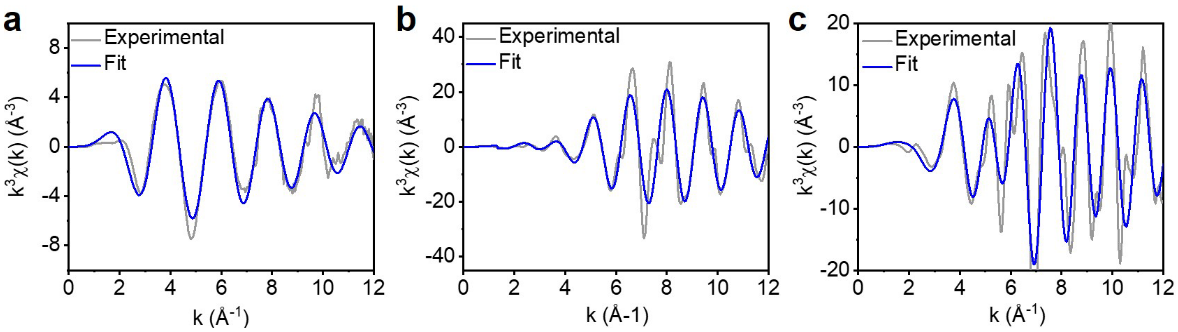

Supplementary Fig. 27 | The EXAFS fitting curves in K-space. The EXAFS fitting curves in K-space for (**a**) NiFe-BTC//G-2h, (**b**) Ni foil, and (**c**) NiO.

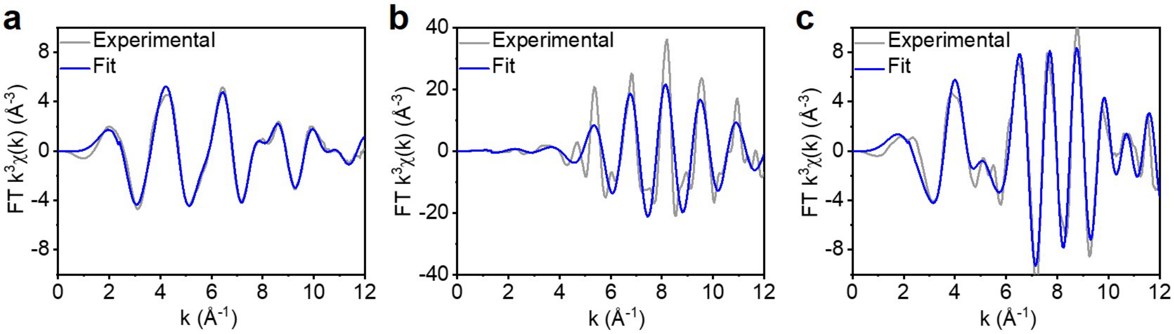

Supplementary Fig. 28 | The EXAFS fitting curves in K-space. The EXAFS fitting curves in K-space for (**a**) NiFe-BTC//G-2h, (**b**) Fe foil, and (**c**) Fe_2O_3.

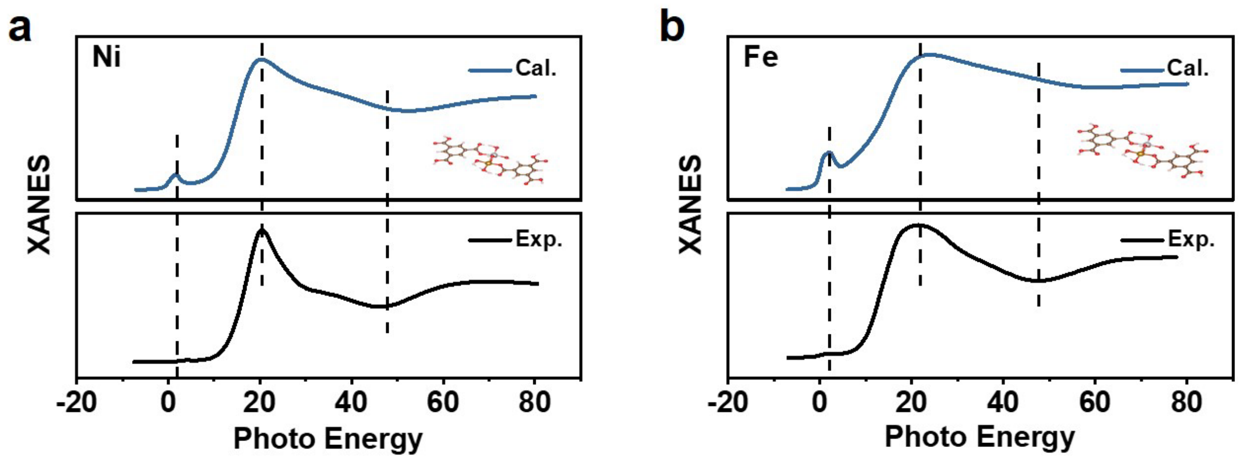

Supplementary Fig. 29 | Comparison between the XANES experimental spectrum and the theoretical spectrum calculated with the structure (NiFe-BTC). The K-edge theoretical XANES simulations are carried out with the FDMNES code in the framework of real-space full multiple-scattering (FMS) scheme. The spectra are convoluted by a Lorentzian function with an energy-dependent width to account for the broadening due to the core-hole and the final state width.

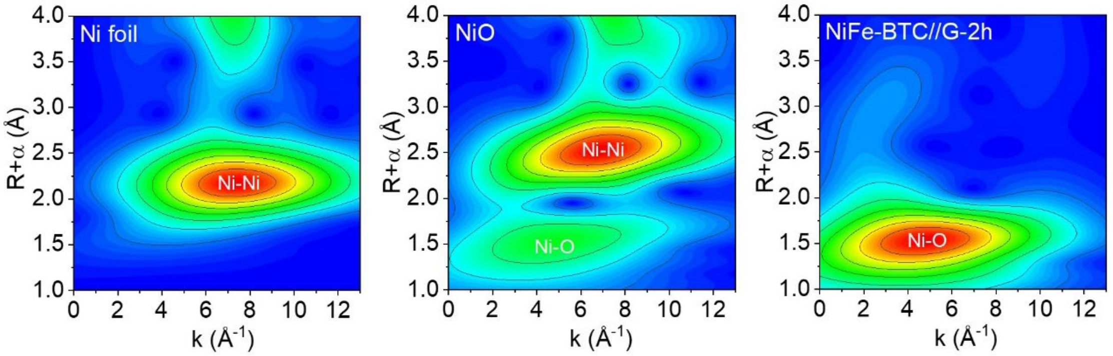

Supplementary Fig. 30 | WT-EXAFS of the Ni foil, NiO, and NiFe-BTC//G. The wavelet transform (WT) analyses of the Ni K-edge displays only one pre-edge peak maximum at ~1.5 Å in the WT-EXAFS map, which is close to that of NiO reference, associated with Ni-O contribution. Compared with the WT plots of Ni foil, the WT signal related to Ni-Ni contribution is not observed in the NiFe-BTC//G. These results further demonstrate that the single Ni atoms coordinated with O atoms, forming the Ni-O bonds.

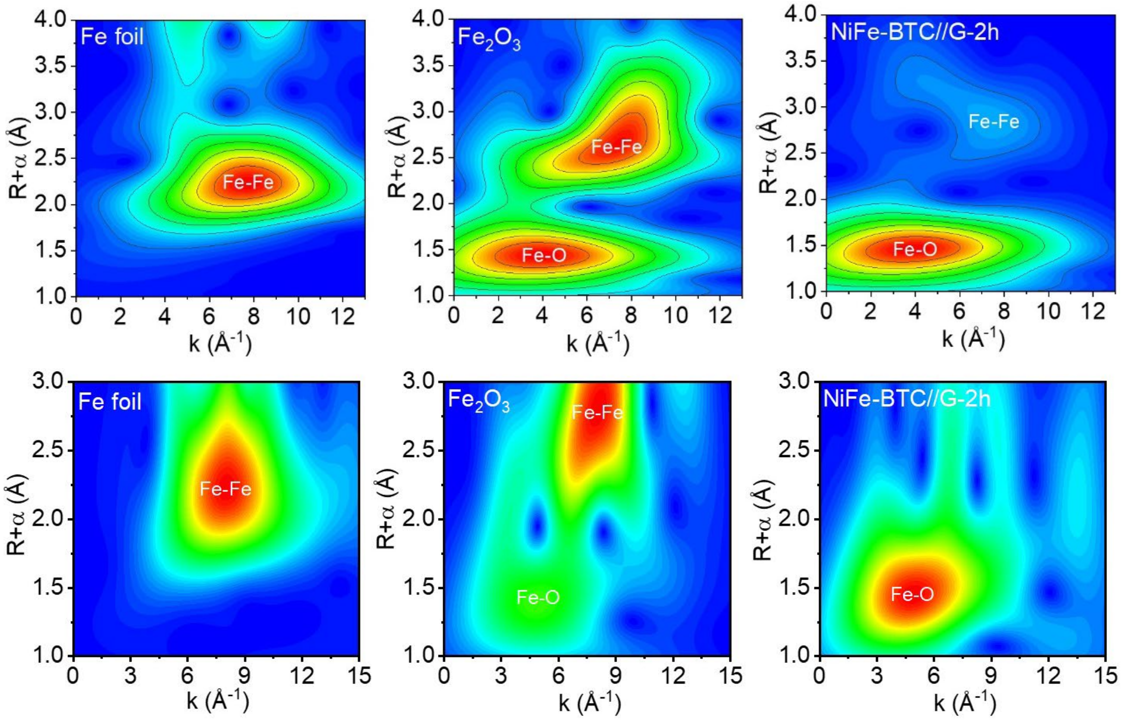

Supplementary Fig. 31 | WT-EXAFS of the Fe foil, Fe_2O_3, and NiFe-BTC//G. Compared with the wavelet transform (WT) plots of Fe foil and Fe_2O_3 reference, only the WT signal related to Fe-O contribution could be observed in the NiFe-BTC//G. These results further demonstrate that the single Fe atoms coordinated with O atoms, forming the Fe-O bonds.

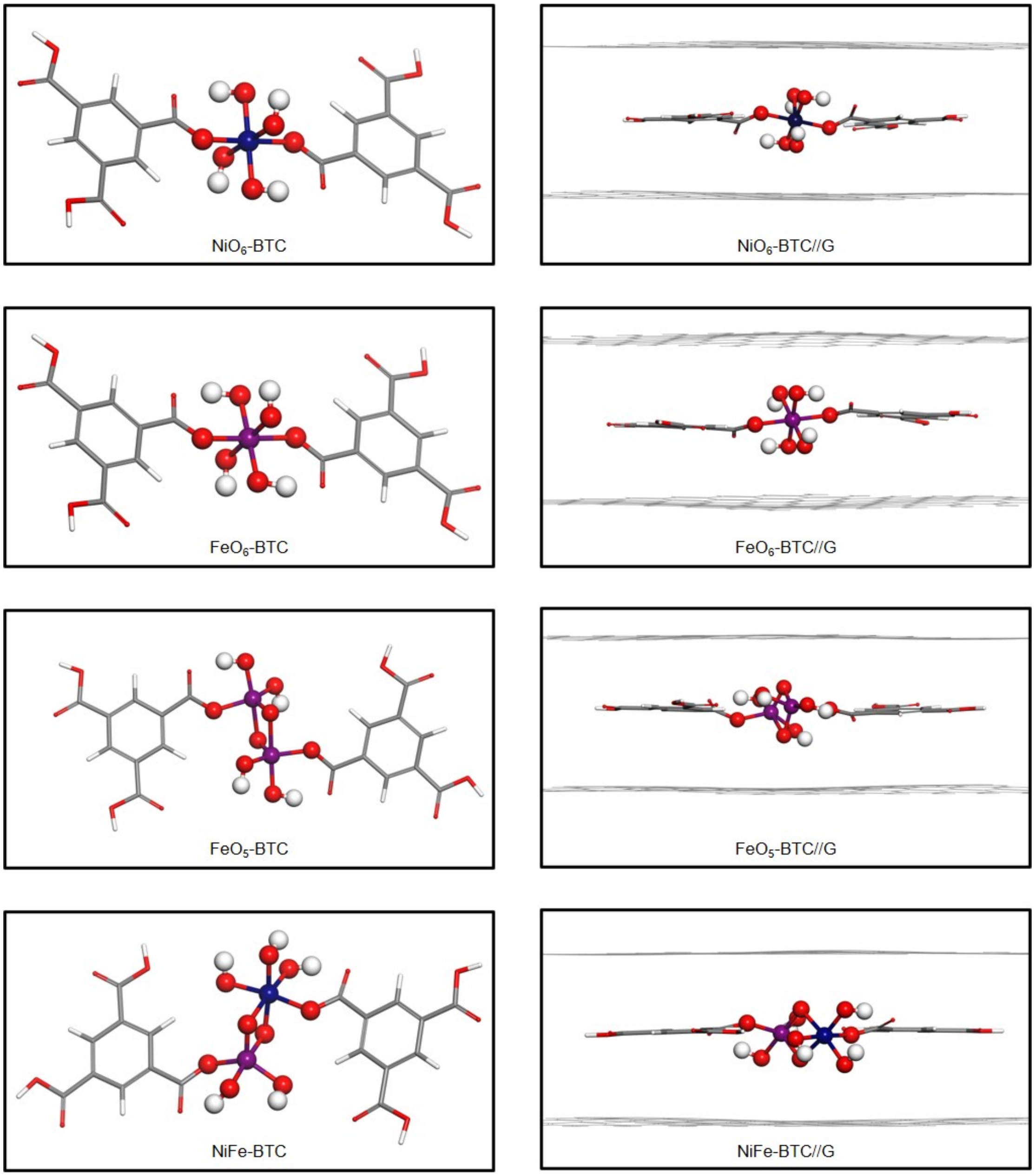

Supplementary Fig. 32 | The structures of pristine and confined MOFs. The subscript number refers to the coordinate number, where NiFe-BTC refers to NiO_6 and FeO_5.

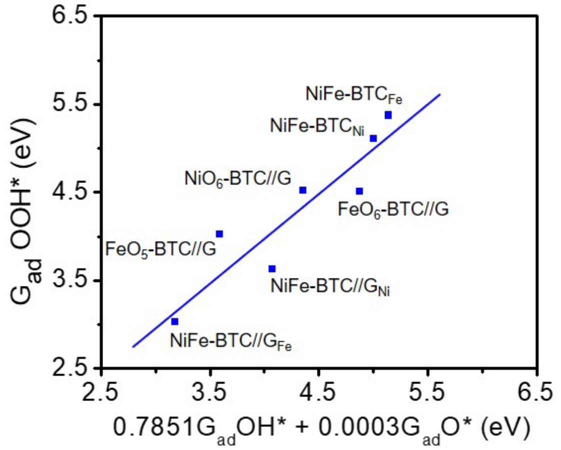

Supplementary Fig. 33 | Two-dimensional linear fitting. The OOH* adsorption energies described by the adsorption energies of O* and OH*.

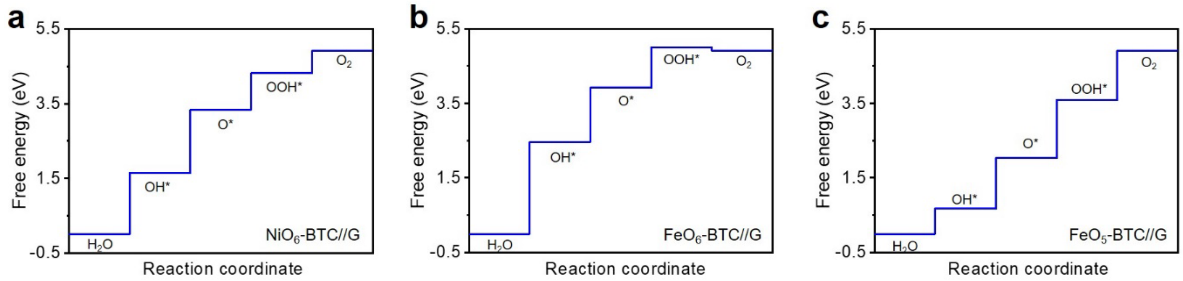

Supplementary Fig. 34 | OER free energy diagrams. OER free energy diagrams on different MOF structures of (**a**) NiO_6-BTC//G, (**b**) FeO_6-BTC//G, and (**c**) FeO_5-BTC//G. The limiting step was found to be OH* deprotonation (1.70 eV), H_2O deprotonation (2.14 eV), and OH* deprotonation (1.40 eV) on NiO_6-BTC//G, FeO_6-BTC//G, and FeO_5-BTC//G, respectively.

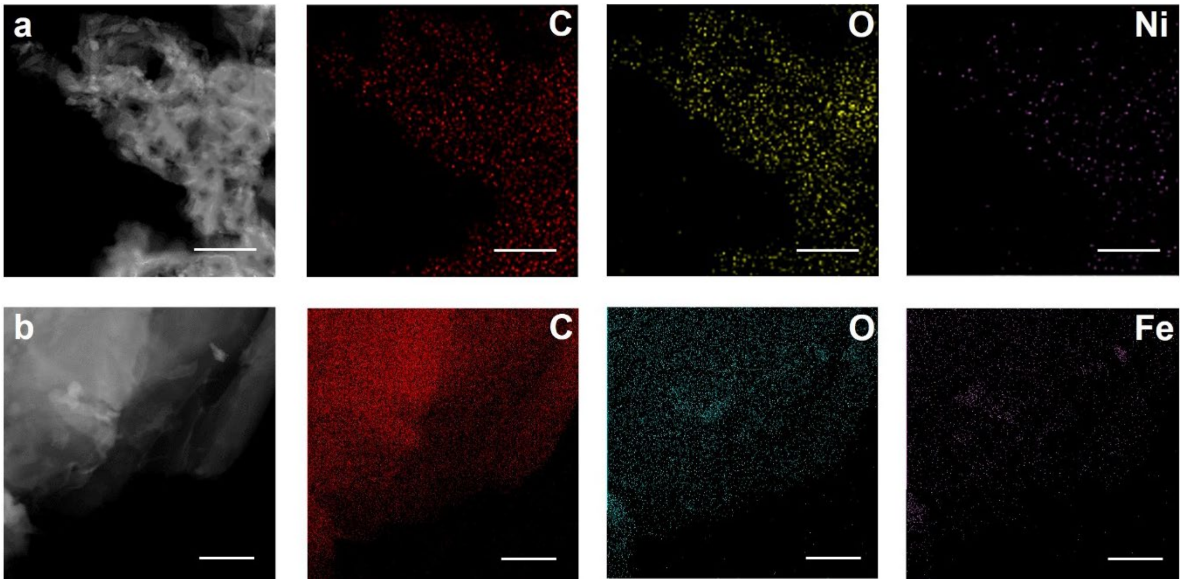

Supplementary Fig. 35 | HAADF-STEM image and EDX element mapping images. HAADF-STEM image and EDX element mapping images of (**a**) Ni-BTC//G-2h and (**b**) Fe-BTC//G-2h. Scale bars, 200 nm (**a**), 500 nm (**b**). The HAADF STEM images and corresponding EDX element (C, O, Fe or Ni) mapping images of Ni-BTC//G-2h and Fe-BTC//G-2h in Supplementary Fig. 35 indicate that the C, O, and transition metal metallic elements (Ni or Fe) are uniformly distributed and confirms that the Ni-BTC//G-2h and Fe-BTC//G-2h electrodes maintain the physical MOF phases.

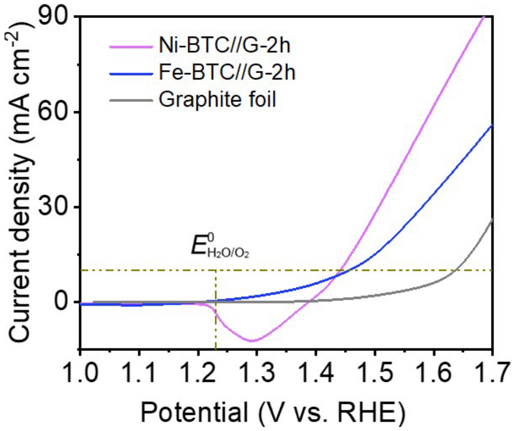

Supplementary Fig. 36 | LSV plots obtained with Ni-BTC//G-2h, Fe-BTC//G-2h, and bare expanded graphite foil for OER at 10 mV s^{-1} in 1.0 M KOH. The Ni-BTC//G-2h and Fe-BTC//G-2h presented the overpotentials of 212 and 226 mV to attain the 10 mA cm^{-2} current density respectively, which were higher than that of NiFe-BTC//G-2h (106 mV at 10 mA cm^{-2}). The better OER performance of NiFe-BTC//G-2h could be attributed to the synergistic effect between Ni and Fe species.

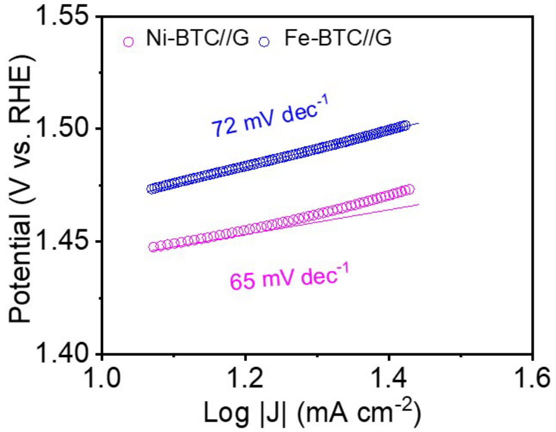

Supplementary Fig. 37 | Tafel plots obtained with Ni-BTC//G-2h and Fe-BTC//G-2h. The OER performances of Ni-BTC//G-2h and Fe-BTC//G-2h were further highlighted by their Tafel slopes of 65 and 72 mV dec^{-1}, which were higher than that of NiFe-BTC//G-2h (55 mV dec^{-1}). The lower Tafel slope of NiFe-BTC//G-2h suggested its favorable catalytic kinetics.

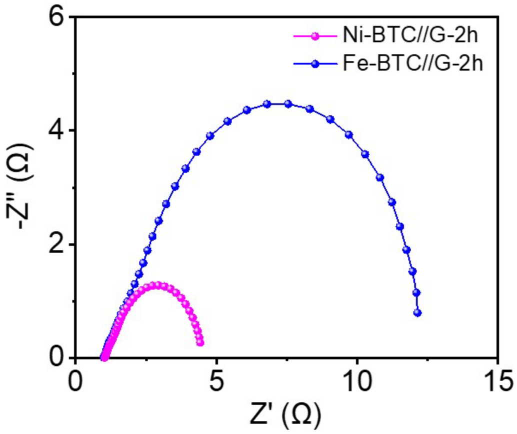

Supplementary Fig. 38 | EIS Nyquist plots obtained with Ni-BTC//G-2h and Fe-BTC//G-2h. The electron transfer abilities for monometallic Ni-BTC/G-2h and Fe-BTC//G-2h are promoted by EIS spectroscopy, where Ni-BTC//G-2h displays much smaller charge transfer resistance (R_{ct}) value of 3.48 Ω compared to Fe-BTC//G-2h (10.37 Ω) (Supplementary Table 3).

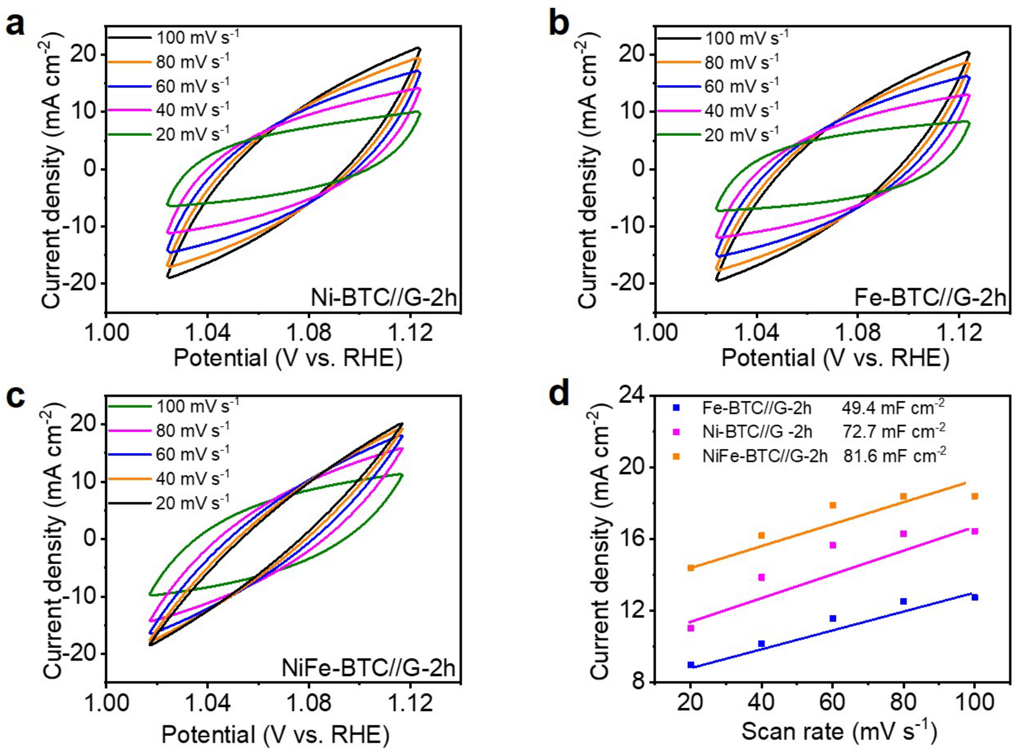

Supplementary Fig. 39 | CV curves and ECSAs. CV curves of (**a**) Ni-BTC//G-2h, (**b**) Fe-BTC//G-2h, and (**c**) NiFe-BTC//G-2h at different scan rates in 1.0 M KOH solution. (**d**) ECSAs of Ni-BTC//G-2h, Fe-BTC//G-2h, and NiFe-BTC//G-2h. The highest C_{dl} value of ~81.6 mF cm^{-2} is observed for NiFe-BTC//G-2h compared to those of Ni-BTC//G-2h (72.7 mF cm^{-2}), Fe-BTC//G-2h (49.4 mF cm^{-2}), revealing that the NiFe-BTC//G-2h electrode possesses highly exposed active sites, much larger than those of monometallic Ni-BTC//G-2h and Fe-BTC//G-2h.

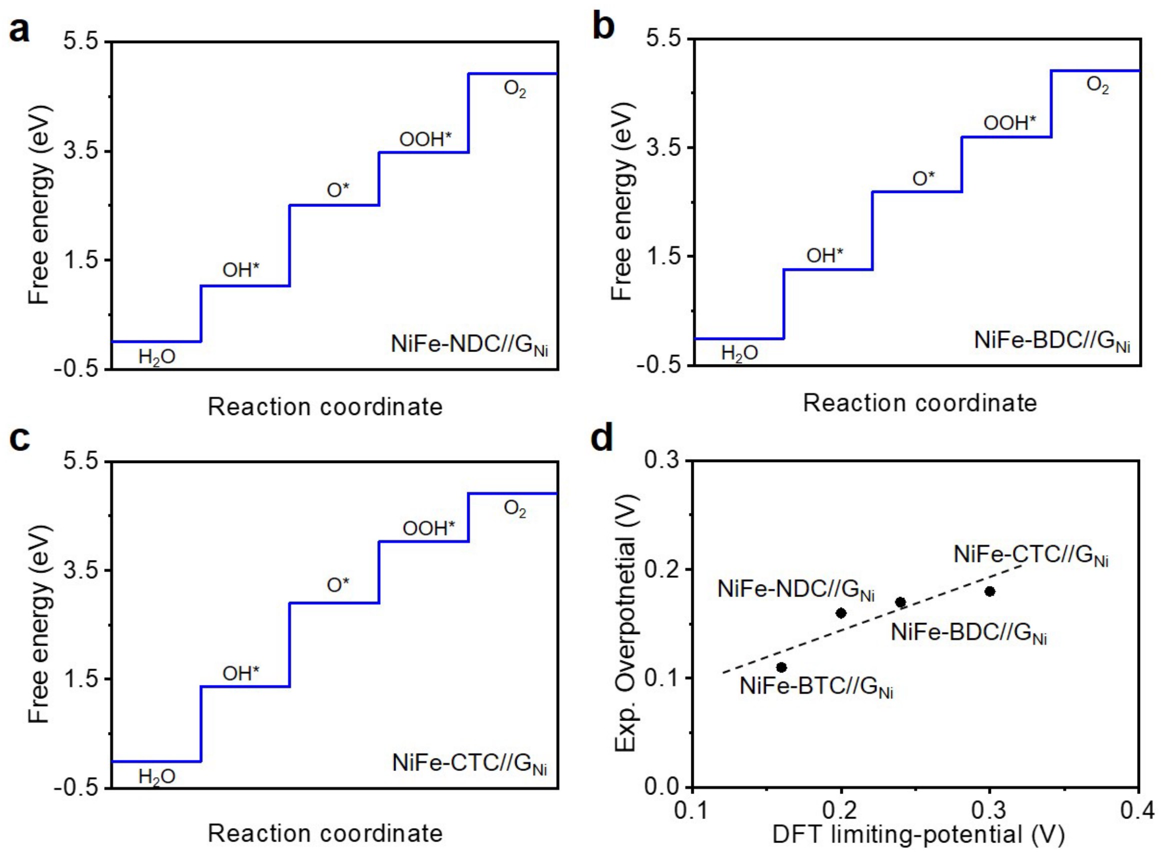

Supplementary Fig. 40 | Free energy diagram of OER. Free energy diagram of OER on (**a**) NiFe-NDC//G_{Ni}, (**b**) NiFe-BDC//G_{Ni}, and (**c**) NiFe-CTC//G_{Ni}. (**d**) The comparison between experimental overpotential and DFT calculated limiting potential for OER with different MOF structures.

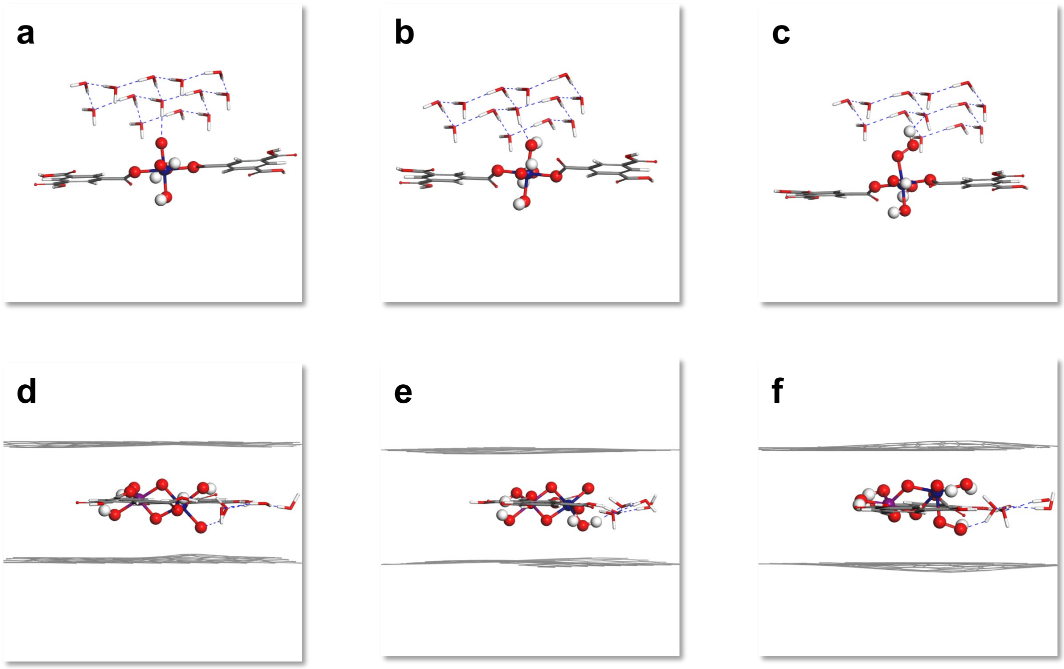

Supplementary Fig. 41 | Explicit water model used for the solvent effect calculation. Explicit water model used for the solvent effect calculation with open system (**a-c** for O*, OH*, and OOH*) and confined system (**d-f** for O*, OH*, and OOH*)

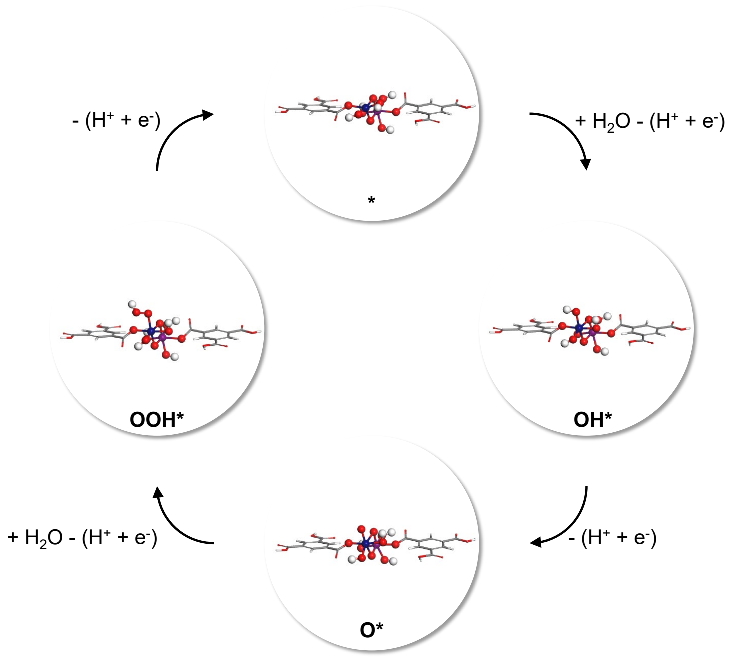

Supplementary Fig. 42 | Schematic diagram of reaction pathway. Schematic diagram of reaction pathway for OER on NiFe-BTC_{Ni} as an example.

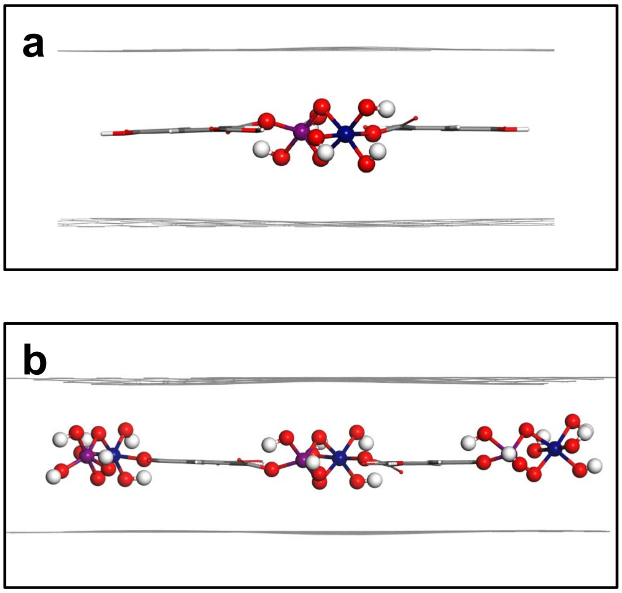

Supplementary Fig. 43 | Three dimensional structures of NiFe-BTC//G. (**a**) Structure of NiFe-BTC//G. (**b**) Structure of NiFe-BTC//G with more NiFe units linked on the other side of BTC structures.

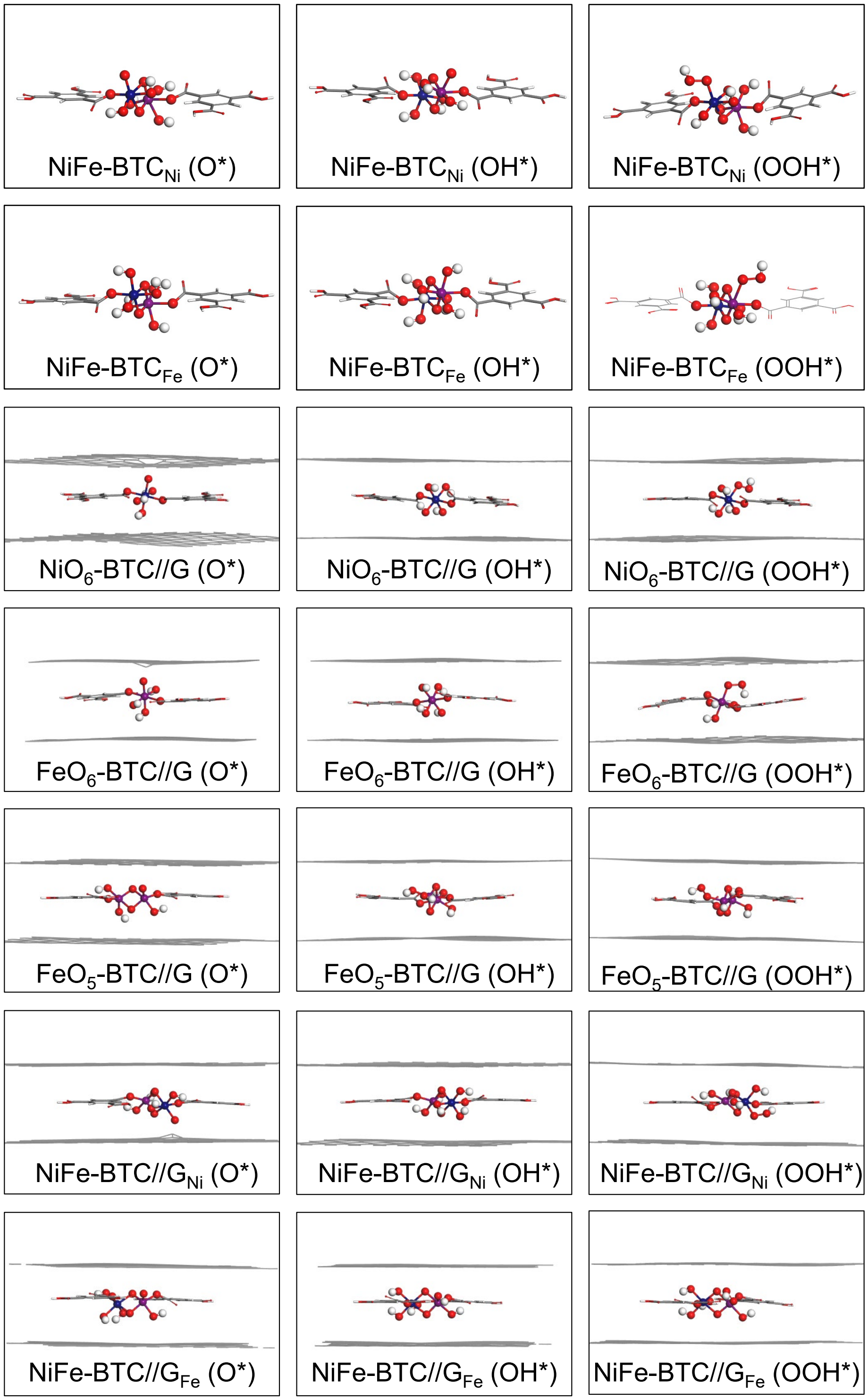

Supplementary Fig. 44 | Optimized adsorption structures. Optimized structures of adsorbed O*, OH* and OOH* on different MOF structures. The subscript of Ni/Fe refer to the active sites for the adsorptions. "//G" refers to the MOF structures in a confinement environment. NiO_6, FeO_6, and FeO_5 refer to the single metal-based MOFs.

Supplementary Tables

Supplementary Table 1. Elemental contents derived from ICP-MS measurement.

Samples	Fe (wt.%)	Ni (wt.%)	Fe:Ni
Bulk NiFe-BTC powder	13.82	0.57	25.48
NiFe-BTC//G-2h	6.56	0.21	32.83

The quantitative measurements by inductively coupled plasma-mass spectrometry (ICP-MS) confirm that NiFe-BTC//G-2h contains 6.56 wt.% of Fe and 0.21 wt.% of Ni, while the bulk NiFe-BTC contains 13.82 wt.% of Fe and 0.57 wt.% of Ni.

Supplementary Table 2. Conductivity measurement of NiFe-BTC.

Thickness (mm)	Pressure (MPa)	Resistivity (GΩ cm)	Conductivity (nS cm^{-1})	Temperature (°C)	Humidity (RH%)
1.76	2	520.00	0.001923077	28	45
1.69	4	378.76	0.002640212	28	45
1.62	6	302.53	0.003305448	28	45
1.58	8	275.51	0.003629681	28	45
1.55	10	246.97	0.004049112	28	45
1.53	12	218.10	0.004584957	28	45
1.51	14	206.49	0.004842848	28	45
1.5	16	193.33	0.005172414	28	45
1.49	18	180.00	0.005555556	28	45
1.48	20	171.42	0.005833662	28	45

Supplementary Table 3. The EIS results of NiFe-BTC//G-2h with other controlled electrodes in 1.0 M KOH solution in this work.

Samples	R_s/Ω	R_{ct}/Ω
NiFe-BTC//G-2h	0.97	0.46
Bulk NiFe-BTC powder	1.79	433.28
Ni-BTC//G-2h	1.25	3.48
Fe-BTC//G-2h	1.29	10.37
Commercial Ir/C//G	1.30	7.13
Commercial RuO_2//G	1.75	8.89

Supplementary Table 4. The EIS results of a series of NiFe-BTC//G-*X* in 1.0 M KOH solution.

Samples	R_s/Ω	R_{ct}/Ω
NiFe-BTC//G-0.5h	0.95	1.23
NiFe-BTC//G-2h	0.97	0.46
NiFe-BTC//G-4h	1.03	0.91
NiFe-BTC//G-2h	1.17	1.36

Supplementary Table 5. Overpotential comparisons of various Ni and/or Fe based MOFs synthesized by our confining strategy with their bulk phases.

Samples	Organic precursors	Overpotentials of bulk MOFs (mV)	Overpotentials of MOFs through our strategy (mV)
NiFe-BTC	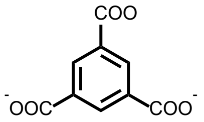	399	106
Ni-BTC		401	212
Fe-BTC		467	226
NiFe-NDC	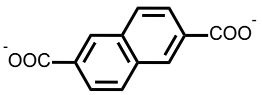	410	170
Ni-NDC		440	225
Fe-NDC		535	240
NiFe-BDC	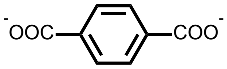	488	163
Fe-BDC		545	203
NiFe-CTC	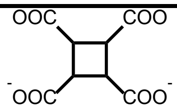	430	181

Supplementary Table 6. The metal mass activities at 1.5 V of various samples.

Samples	Loading amount (mg cm^{-2})	Metal mass activity at 1.5 V
NiFe-BTC//G-2h	13.82	111.643 A $g^{-1}_{Ni,Fe}$
Bulk NiFe-BTC powder	1.0	9.138 A $g^{-1}_{Ni,Fe}$
Ir/C//G	1.0	82.168 A g^{-1}_{Ir}
RuO_2//G	1.0	13.603 A g^{-1}_{Ru}

As shown in Supplementary Table 3, the Ir/C//G obtains the metal mass activity of 82.168 A g^{-1}_{Ir} at 1.5 V, which was higher than that of RuO_2//G (13.603 A g^{-1}_{Ru}). This could be attributed to the promotional role of expanded graphite foil and the conversion of metallic Ir to hydrous Ir oxide.

Supplementary Table 7. Comparison of electrocatalytic OER performances of NiFe-BTC//G-2h with other reported MOF-based and transition metal based OER electrocatalysts in alkaline media.

Catalyst	Electrolyte	Overpotential @10 mA cm^{-2} (mV)	Tafel slope (mV dec^{-1})	Ref.
NiS_x/C_3N_4	1.0 M KOH	32	48	22
CoMnP-NPC/CC	1.0 M KOH	42	54.26	23
$Ni_2P/(NiFe)_2P(O)$ NAs	1.0 M KOH	150	60	24
Ir_1/NFS	1.0 M KOH	170	33	25
NiFeCP/NF	1.0 M KOH	188	29	26
$Fe_{0.38}Ni_{0.62}$-MOF	1.0 M KOH	190	57.4	27
NiFc-MOF/NF	1.0 M KOH	195	48.5	28
NiFe-MOF/FeCH-NF	1.0 M KOH	200	51.3	29
WC_x-FeNi	1.0 M KOH	211	56	30
Ni-Fe-MOF NSs	1.0 M KOH	221	56	31
NiFe-NFF	1.0 M KOH	227	38.9	32
$Co_{0.85}Se_{1-x}$@C	1.0 M KOH	231	57	33
NiFe-MOF/NF	0.1 M KOH	240	34	34
$UCoO_4$	1.0 M KOH	250	47	35
Fe1Ni2-BDC	1.0 M KOH	260	35	36
NiFe-UMNs	1.0 M KOH	260	30	37
Ni_3N-NiMoN	1.0 M KOH	277	64	38
NiCoFeP/C	1.0 M KOH	270	65	39
NFN-MOF/NF	1.0 M KOH	240	58.8	40
$NiCo/Fe_3O_4$/MOF-74	1.0 M KOH	238	29	41
Ni_2P-CoP	0.1 M KOH	320	69	42
NiFe-MOF-74	1.0 M KOH	223	71.6	43
CoBDC-Fc-NF	1.0 M KOH	178	51	44
$NiMoO_x$/NiMoS	1.0 M KOH	186	34	45
$(Ni,Co)_{0.85}Se$@NiCo-LDH	1.0 M KOH	216	77	46
NiCo-UMOFNs/Cu foam	1.0 M KOH	189	42	47
Fe/Ni-BTC@NF	0.1 M KOH	270	47	48
Ni@NC	1.0 M KOH	280	45	49
$MOF(Fe_1-Co_3)_{550N}$	0.1 M KOH	390	72.9	50
NiFe LDH/GO	1.0 M KOH	230	42	51

NiFe LDH	1.0 M KOH	300	40	[52]
NiFeOOH	1.0 M KOH	340	60	[53]
$Ir_{0.1}/Ni_9Fe$ SAC	1.0 M KOH	183	49	[54]
Ru SAs/AC-FeCoNi	1.0 M KOH	205	40	[55]
$(Ru\text{-}Co)O_x$-350	1.0 M KOH	265	60	[56]
α-Li_2IrO_3	0.1 M KOH	290	50	[57]
**NiFe-BTC//G-2h**	**1.0 M KOH**	**106**	**55**	**This work**

Supplementary Table 8. EXAFS fitting parameters at the Fe and Ni K-edge (S_0^2 = 0.71 and 0.78) of as-prepared samples in this work.

Sample	Path	C.N.	R (Å)	$\sigma^2 \times 10^{-3}$ (Å^2)	ΔE_0 (eV)	R factor
Fe foil	Fe-Fe	8*	2.47±0.01	4.7±0.9	6.8±1.3	0.001
	Fe-Fe	6*	2.84±0.01	5.9±1.7	5.2±2.6	
Fe_2O_3	Fe-O	6.2±2.0	1.98±0.03	14.0±4.3	-3.9±3.6	0.017
	Fe-Fe	5.1±1.4	2.97±0.02	7.1±2.3	1.2±2.5	
	Fe-Fe	1.7±0.8	3.46±0.02	10.2±3.0	8.9±1.8	
NiFe-BTC//G (Fe)	Fe-O	5.0±0.2	2.02±0.01	3.6±0.9	1.08±1.0	0.0163
Ni foil	Ni-Ni	12*	2.48±0.01	6.0±0.2	6.9±0.4	0.001
NiO	Ni-O	5.8±0.9	2.09±0.01	5.6±1.4	-3.0±1.8	0.004
	Ni-Ni	12.6±1.1	2.95±0.01	6.3±0.5	-5.4±0.9	
NiFe-BTC//G (Ni)	Ni-O	6.1±0.5	2.06±0.01	6.3±0.8	-4.2±0.9	0.007

[a]*C.N.*: coordination numbers; [b]*R*: bond distance; [c]σ^2: Debye-Waller factors; [d]ΔE_0: the inner potential correction. *R* factor: goodness of fit. *the experimental EXAFS fit of metal foil by fixing CN as the known crystallographic value.

The obtained XAFS data was processed in Athena (version 0.9.25) for background, pre-edge line and post-edge line calibrations. Then Fourier transform fitting was carried out in Artemis (version 0.9.25). The k^3 weighting, k-range of 2 - 12 Å^{-1} and R range of 1 - ~3 Å were used for the fitting. The four parameters, coordination number, bond length, Debye-Waller factor and E_0 shift (CN, R, σ^2, ΔE_0) were fitted without anyone was fixed, constrained, or correlated. For Wavelet Transform analysis, the $\chi(k)$ exported from Athena was imported into the Hama Fortran code. The parameters were listed as follow: R range, 1 - 4 Å, k range, 0 - 12 Å^{-1}; k weight, 2; and Morlet function with κ=10, σ=1 was used as the mother wavelet to provide the overall distribution.

Supplementary Table 9. Adsorption free energy of O*, OH* and OOH* on different MOFs.

Adsorption free energy (eV)*	O*	OH*	OOH*
NiFe-BTC_{Ni}	4.89	2.30	4.69
NiFe-BTC_{Fe}	5.75	2.64	5.21
NiO_6-BTC//G	3.34	1.64	4.52
FeO_6-BTC//G	3.92	2.46	5.11
NiFe-BTC//G_{Ni}	2.68	1.28	3.63
FeO_5-BTC//G	2.03	0.67	4.02
NiFe-BTC//G_{Fe}	1.12	0.14	3.03

*Adsorption energies were calculated with the gas references of H_2 and H_2O. All the energies were correct to free energy with the consideration of solvent effect.

Supplementary Table 10. Accuracy tests (reaction free energy at 1.23 V vs RHE) of cut-off energy and force convergency on reaction free energy of NiFe-BTC//G_{Ni}.

	Original	Cut-off energy test		Force convergency test	
Cut-off energy (eV)	400	450		400	
Force convergency (eV·$Å^{-1}$)	0.05	0.05		0.01	
	Reaction energy (eV)	Reaction energy (eV)	Error (eV)	Reaction energy (eV)	Error (eV)
$H_2O \rightarrow (H^+ + e^-) + OH^*$	0.05	0.06	0.01	0.04	-0.01
$OH^* \rightarrow (H^+ + e^-) + O^*$	**0.17***	**0.19**	0.02	**0.18**	0.01
$O^* + H_2O \rightarrow (H^+ + e^-) + OOH^*$	-0.28	-0.29	-0.01	-0.28	0.00
$OOH^* \rightarrow (H^+ + e^-) + O_2 + *$	0.06	0.04	-0.02	0.06	0.00

* The bold numbers refer to the limiting-energies, namely DFT calculated limiting potential.

Supplementary Table 11. Solvent effect (eV) by implicit/explicit models on either open or confined systems.

	Implicit model on open system	Explicit model on open system	Implicit model on confined system	Explicit model on confined system
O*	0.12	0.09	0.14	0.18
OH*	0.14	0.12	0.14	0.20
OOH*	0.42	0.40	0.34	0.24

It is found that, the solvent effect on the open system using implicit model was confirmed to show its reliability by the explicit model, displaying an error less than 0.03 eV. For the solvent effect for the confined system, a similar energy change was found on O* and OH* with also a different less than 0.02 eV compared with that on open system, while larger difference was found for that on OOH*. It may be caused by the larger size of OOH*, which was more affected in the confinement environment. Moreover, an error of around 0.1 eV was obtained on the solvent effect using implicit or explicit model in the confinement effect, which was caused by the local water structures for the explicit model.

Supplementary Table 12. Test of energy effect from spin magnetization on NiFe-BTC//G_{Ni}.

	Reaction energy (eV) Spin-off	Reaction energy (eV) Spin-on
$H_2O \rightarrow (H^+ + e^-) + OH^*$	0.05	0.07
$OH^* \rightarrow (H^+ + e^-) + O^*$	**0.17***	**0.21**
$O^* + H_2O \rightarrow (H^+ + e^-) + OOH^*$	-0.28	-0.14
$OOH^* \rightarrow (H^+ + e^-) + O_2 + *$	0.06	-0.14

* The bold numbers refer to the limiting-energies, namely DFT calculated limiting potential.

Supplementary Table 13. Test of energy effect from varying NiFe units linked with BTC on NiFe-BTC//G_{Ni}.

	Reaction energy (eV) One NiFe unit	Reaction energy (eV) Three NiFe units
$H_2O \rightarrow (H^+ + e^-) + OH^*$	0.05	-0.01
$OH^* \rightarrow (H^+ + e^-) + O^*$	**0.17***	**0.19**
$O^* + H_2O \rightarrow (H^+ + e^-) + OOH^*$	-0.28	-0.23
$OOH^* \rightarrow (H^+ + e^-) + O_2 + *$	0.06	0.05

* The bold numbers refer to the limiting-energies, namely DFT calculated limiting potential.

Supplementary References

1 Funke, H., Chukalina, M. & Scheinost, A. C. A new FEFF-based wavelet for EXAFS data analysis. *J Synchrotron Radiat* **14**, 426-432, doi:10.1107/S0909049507031901 (2007).

2 Ravel, B. & Newville, M. ATHENA, ARTEMIS, HEPHAESTUS: data analysis for X-ray absorption spectroscopy using IFEFFIT. *J Synchrotron Radiat* **12**, 537-541, doi:10.1107/S0909049505012719 (2005).

3 <33333333333333333333.pdf>.

4 Cao, X.-M., Burch, R., Hardacre, C. & Hu, P. An understanding of chemoselective hydrogenation on crotonaldehyde over Pt(111) in the free energy landscape: The microkinetics study based on first-principles calculations. *Catalysis Today* **165**, 71-79, doi:10.1016/j.cattod.2010.12.056 (2011).

5 Nørskov, J. K. *et al.* Origin of the Overpotential for Oxygen Reduction at a Fuel-Cell Cathode. *J. Phys. Chem. B* **108**, 17886-17892, doi:10.1007/s40592-020-00107-z (2004).

6 Calle-Vallejo, F., Martínez, J. I., García-Lastra, J. M., Abad, E. & Koper, M. T. M. Oxygen reduction and evolution at single-metal active sites: Comparison between functionalized graphitic materials and protoporphyrins. *Surface Science* **607**, 47-53, doi:10.1016/j.susc.2012.08.005 (2013).

7 Sha, Y., Yu, T. H., Liu, Y., Merinov, B. V. & Goddard, W. A. Theoretical Study of Solvent Effects on the Platinum-Catalyzed Oxygen Reduction Reaction. *The Journal of Physical Chemistry Letters* **1**, 856-861, doi:10.1021/jz9003153 (2010).

8 Mathew, K., Kolluru, V. S. C., Mula, S., Steinmann, S. N. & Hennig, R. G. Implicit self-consistent electrolyte model in plane-wave density-functional theory. *J. Chem. Phys.* **151**, 234101 (2019).

9 Mathew, K., Sundararaman, R., Letchworth-Weaver, K., Arias, T. A. & Hennig, R. G. Implicit solvation model for density-functional study of nanocrystal surfaces and reaction pathways. *J Chem Phys* **140**, 084106, doi:10.1063/1.4865107 (2014).

10 Wu, J. B., Lin, M. L., Cong, X., Liu, H. N. & Tan, P. H. Raman spectroscopy of graphene-based materials and its applications in related devices. *Chem Soc Rev* **47**, 1822-1873, doi:10.1039/c6cs00915h (2018).

11 Jahan, M., Bao, Q. & Loh, K. P. Electrocatalytically active graphene-porphyrin MOF composite for oxygen reduction reaction. *J Am Chem Soc* **134**, 6707-6713, doi:10.1021/ja211433h (2012).

12 Thangavel, P. *et al.* Graphene-nanoplatelets-supported NiFe-MOF: high-efficiency and ultra-stable oxygen electrodes for sustained alkaline anion exchange membrane water electrolysis. *Energy & Environmental Science* **13**, 3447-3458, doi:10.1039/d0ee00877j (2020).

13 Louie, M. W. & Bell, A. T. An investigation of thin-film Ni-Fe oxide catalysts for the electrochemical evolution of oxygen. *J Am Chem Soc* **135**, 12329-12337, doi:10.1021/ja405351s (2013).

14 Cai, L. *et al.* One-Step Hydrothermal Deposition of Ni:FeOOH onto Photoanodes for Enhanced Water Oxidation. *ACS Energy Letters* **1**, 624-632, doi:10.1021/acsenergylett.6b00303 (2016).

15 Nguyen, L. T. L., Nguyen, T. T., Nguyen, K. D. & Phan, N. T. S. Metal–organic framework MOF-199 as an efficient heterogeneous catalyst for the aza-Michael reaction. *Applied Catalysis A: General* **425-426**, 44-52, doi:10.1016/j.apcata.2012.02.045 (2012).

16 Kong, L., Zhu, J., Shuang, W. & Bu, X.-H. Nitrogen-Doped Wrinkled Carbon Foils Derived from MOF Nanosheets for Superior Sodium Storage. *Advanced Energy Materials* **8**, doi:10.1002/aenm.201801515 (2018).

17 Zhao, K. *et al.* Vertically aligned MnO2 nanosheets coupled with carbon nanosheets derived from Mn-MOF nanosheets for supercapacitor electrodes. *Journal of Materials Science* **53**, 13111-13125, doi:10.1007/s10853-018-2562-3 (2018).

18 Li, S. *et al.* Transition metal-based bimetallic MOFs and MOF-derived catalysts for electrochemical oxygen evolution reaction. *Energy & Environmental Science* **14**, 1897-1927, doi:10.1039/d0ee03697h (2021).

19 Du, J., Li, F. & Sun, L. Metal-organic frameworks and their derivatives as electrocatalysts for the oxygen evolution reaction. *Chem Soc Rev* **50**, 2663-2695, doi:10.1039/d0cs01191f (2021).

20 Suen, N. T. *et al.* Electrocatalysis for the oxygen evolution reaction: recent development and future perspectives. *Chem Soc Rev* **46**, 337-365, doi:10.1039/c6cs00328a (2017).

21 Xiong, B., Chen, L. & Shi, J. Anion-Containing Noble-Metal-Free Bifunctional Electrocatalysts for Overall Water Splitting. *ACS Catalysis* **8**, 3688-3707, doi:10.1021/acscatal.7b04286 (2018).

22 Zahran, Z. N. *et al.* Electrocatalytic water splitting with unprecedentedly low overpotentials by nickel sulfide

nanowires stuffed into carbon nitride scabbards. *Energy & Environmental Science* **14**, 5358-5365, doi:10.1039/d1ee00509j (2021).
23 Xu, Y. *et al.* Amorphous Carbon Interconnected Ultrafine CoMnP with Enhanced Co Electron Delocalization Yields Pt‐Like Activity for Alkaline Water Electrolysis. *Advanced Functional Materials*, doi:10.1002/adfm.202112623 (2022).
24 Xi, W. *et al.* Oxygen-Doped Nickel Iron Phosphide Nanocube Arrays Grown on Ni Foam for Oxygen Evolution Electrocatalysis. *Small* **14**, e1802204, doi:10.1002/smll.201802204 (2018).
25 Lei, Z. *et al.* Coordination modulation of iridium single-atom catalyst maximizing water oxidation activity. *Nat Commun* **13**, 24, doi:10.1038/s41467-021-27664-z (2022).
26 Li, W. *et al.* A bio-inspired coordination polymer as outstanding water oxidation catalyst via second coordination sphere engineering. *Nat Commun* **10**, 5074, doi:10.1038/s41467-019-13052-1 (2019).
27 Wan, Z. *et al.* Oxygen-Evolution Catalysts Based on Iron-Mediated Nickel Metal–Organic Frameworks. *ACS Applied Nano Materials* **2**, 6334-6342, doi:10.1021/acsanm.9b01330 (2019).
28 Liang, J. *et al.* Ferrocene-Based Metal-Organic Framework Nanosheets as a Robust Oxygen Evolution Catalyst. *Angew Chem Int Ed Engl* **60**, 12770-12774, doi:10.1002/anie.202101878 (2021).
29 Du, J., Xu, S., Sun, L. & Li, F. Iron carbonate hydroxide templated binary metal-organic frameworks for highly efficient electrochemical water oxidation. *Chem Commun (Camb)* **55**, 14773-14776, doi:10.1039/c9cc07433c (2019).
30 Li, S. *et al.* Oxygen-evolving catalytic atoms on metal carbides. *Nat Mater* **20**, 1240-1247, doi:10.1038/s41563-021-01006-2 (2021).
31 Li, F. L. *et al.* Large-Scale, Bottom-Up Synthesis of Binary Metal-Organic Framework Nanosheets for Efficient Water Oxidation. *Angew Chem Int Ed Engl* **58**, 7051-7056, doi:10.1002/anie.201902588 (2019).
32 Cao, C., Ma, D. D., Xu, Q., Wu, X. T. & Zhu, Q. L. Semisacrificial Template Growth of Self‐Supporting MOF Nanocomposite Electrode for Efficient Electrocatalytic Water Oxidation. *Advanced Functional Materials* **29**, doi:10.1002/adfm.201807418 (2018).
33 Zhang, L. *et al.* Selenic Acid Etching Assisted Vacancy Engineering for Designing Highly Active Electrocatalysts toward the Oxygen Evolution Reaction. *Adv Mater* **33**, e2007523, doi:10.1002/adma.202007523 (2021).
34 Duan, J., Chen, S. & Zhao, C. Ultrathin metal-organic framework array for efficient electrocatalytic water splitting. *Nat Commun* **8**, 15341, doi:10.1038/ncomms15341 (2017).
35 Lin, X. *et al.* 5f Covalency Synergistically Boosting Oxygen Evolution of UCoO4 Catalyst. *J Am Chem Soc* **144**, 416-423, doi:10.1021/jacs.1c10311 (2022).
36 Li, J. *et al.* Low-Crystalline Bimetallic Metal–Organic Framework Electrocatalysts with Rich Active Sites for Oxygen Evolution. *ACS Energy Letters* **4**, 285-292, doi:10.1021/acsenergylett.8b02345 (2018).
37 Hai, G. *et al.* High-performance oxygen evolution catalyst using two-dimensional ultrathin metal-organic frameworks nanosheets. *Nano Energy* **44**, 345-352, doi:10.1016/j.nanoen.2017.11.071 (2018).
38 Wu, A. *et al.* Integrating the active OER and HER components as the heterostructures for the efficient overall water splitting. *Nano Energy* **44**, 353-363, doi:10.1016/j.nanoen.2017.11.045 (2018).
39 Wei, X. *et al.* Carbon-incorporated porous honeycomb NiCoFe phosphide nanospheres derived from a MOF precursor for overall water splitting. *Chem Commun (Camb)* **55**, 10896-10899, doi:10.1039/c9cc05225a (2019).
40 Senthil Raja, D., Chuah, X.-F. & Lu, S.-Y. In Situ Grown Bimetallic MOF-Based Composite as Highly Efficient Bifunctional Electrocatalyst for Overall Water Splitting with Ultrastability at High Current Densities. *Advanced Energy Materials* **8**, doi:10.1002/aenm.201801065 (2018).
41 Wang, X. *et al.* Constructing NiCo/Fe3O4 Heteroparticles within MOF-74 for Efficient Oxygen Evolution Reactions. *J Am Chem Soc* **140**, 15336-15341, doi:10.1021/jacs.8b08744 (2018).
42 Liang, X. *et al.* MOF-Derived Formation of Ni2P-CoP Bimetallic Phosphides with Strong Interfacial Effect toward Electrocatalytic Water Splitting. *ACS Appl Mater Interfaces* **9**, 23222-23229, doi:10.1021/acsami.7b06152 (2017).
43 Xing, J. *et al.* In situ growth of well-ordered NiFe-MOF-74 on Ni foam by Fe(2+) induction as an efficient and stable electrocatalyst for water oxidation. *Chem Commun (Camb)* **54**, 7046-7049, doi:10.1039/c8cc03112f (2018).
44 Xue, Z. *et al.* Missing-linker metal-organic frameworks for oxygen evolution reaction. *Nat Commun* **10**, 5048,

doi:10.1038/s41467-019-13051-2 (2019).
45 Zhai, P. *et al.* Engineering active sites on hierarchical transition bimetal oxides/sulfides heterostructure array enabling robust overall water splitting. *Nat Commun* **11**, 5462, doi:10.1038/s41467-020-19214-w (2020).
46 Xia, C., Jiang, Q., Zhao, C., Hedhili, M. N. & Alshareef, H. N. Selenide-Based Electrocatalysts and Scaffolds for Water Oxidation Applications. *Adv Mater* **28**, 77-85, doi:10.1002/adma.201503906 (2016).
47 Zhao, S. *et al.* Ultrathin metal–organic framework nanosheets for electrocatalytic oxygen evolution. *Nature Energy* **1**, doi:10.1038/nenergy.2016.184 (2016).
48 Wang, L. *et al.* Fe/Ni Metal-Organic Frameworks and Their Binder-Free Thin Films for Efficient Oxygen Evolution with Low Overpotential. *ACS Appl Mater Interfaces* **8**, 16736-16743, doi:10.1021/acsami.6b05375 (2016).
49 Xu, Y. *et al.* Nickel Nanoparticles Encapsulated in Few-Layer Nitrogen-Doped Graphene Derived from Metal-Organic Frameworks as Efficient Bifunctional Electrocatalysts for Overall Water Splitting. *Adv Mater* **29**, doi:10.1002/adma.201605957 (2017).
50 Han, Y., Zhai, J., Zhang, L. & Dong, S. Direct carbonization of cobalt-doped NH2-MIL-53(Fe) for electrocatalysis of oxygen evolution reaction. *Nanoscale* **8**, 1033-1039, doi:10.1039/c5nr06626c (2016).
51 Wei Ma, R. M., Chengxiang Wang, Jianbo Liang, Xiaohe Liu, Kechao Zhou, Takayoshi Sasaki. A Superlattice of Alternately Stacked Ni-Fe Hydroxide Nanosheets and Graphene for Efficient Splitting ofWater. *ACS Nano* **9**, 1977-1984 (2015).
52 Song, F. & Hu, X. Exfoliation of layered double hydroxides for enhanced oxygen evolution catalysis. *Nat Commun* **5**, 4477, doi:10.1038/ncomms5477 (2014).
53 Swierk, J. R., Klaus, S., Trotochaud, L., Bell, A. T. & Tilley, T. D. Electrochemical Study of the Energetics of the Oxygen Evolution Reaction at Nickel Iron (Oxy)Hydroxide Catalysts. *The Journal of Physical Chemistry C* **119**, 19022-19029, doi:10.1021/acs.jpcc.5b05861 (2015).
54 Zheng, X. *et al.* Origin of enhanced water oxidation activity in an iridium single atom anchored on NiFe oxyhydroxide catalyst. *Proc Natl Acad Sci U S A* **118**, doi:10.1073/pnas.2101817118 (2021).
55 Hu, Y. *et al.* Single Ru Atoms Stabilized by Hybrid Amorphous/Crystalline FeCoNi Layered Double Hydroxide for Ultraefficient Oxygen Evolution. *Advanced Energy Materials* **11**, doi:10.1002/aenm.202002816 (2020).
56 Wang, C. *et al.* Ultralow Ru doping induced interface engineering in MOF derived ruthenium-cobalt oxide hollow nanobox for efficient water oxidation electrocatalysis. *Chemical Engineering Journal* **420**, doi:10.1016/j.cej.2021.129805 (2021).
57 Yang, C. *et al.* Cation insertion to break the activity/stability relationship for highly active oxygen evolution reaction catalyst. *Nat Commun* **11**, 1378, doi:10.1038/s41467-020-15231-x (2020).
