# Supplementary material for: Exceptional catalytic activity of oxygen evolution reaction via two-dimensional graphene multilayer confined metal-organic frameworks
Source: Nat Commun. 2022 Oct 18;13:6171. doi: 10.1038/s41467-022-33847-z (PMC9579180; doi:10.1038/s41467-022-33847-z)
Supplement: Supplementary file 3 — Description of Additional Supplementary Files [file 41467_2022_33847_MOESM3_ESM.pdf]

Description of Additional Supplementary Files

File Name: Supplementary Movie 1
Description: The Supplementary Movie 1 shows the preparation of NiFe-BTC//G-2h. The synthetic process concludes 3 steps.In the first step, an electrochemical exfoliation process is conducted by anodization of graphite foil in dilute sulfuric acid solution (0.5 M, 50 mL) with using Pt foil (10 × 10 mm) as counter electrode and graphite foil (10 × 30 mm) as working electrode under 3.0 V for 30 min. The exfoliated graphite foil is rinsed with DI-water several times and dried in oven at 60 ºC. Secondly, 420 mg of 1,3,5-benzenetricarboxylic acid and 240 mg of NaOH are dissolved in DI-water (75 mL) and rigorously stir for 30 min to obtain transparent solution. Then, the dilated graphite foil is put into the above solution and intercalated by 1,3,5-benzenetricarboxylic group under 5.0 V for 2h. The as-obtained graphite foil is immersed into DI-water and slightly sonicated at an ultrasonic frequency of 20 KHz for 30 min to remove the excessive organic 1,3,5-benzenetricarboxylic trisodium salt on the surface of graphite foil as well as small pieces of deciduous graphite flakes. Finally, 713 mg of $NiCl_2·6H_2O$ and 1.21 g of $Fe(NO_3)_3·9H_2O$ are dissolved in a mixed solvent of DI-water and ethanol (100 mL, V:V=1:1) and stirred for 20 min to form transparent inorganic salt solution. The treated graphite is immersed into the above solution for 24 h, during which the NiFe-BTC is in situ fabricated. The as-prepared electrode (denoted as NiFe-BTC//G-2h) is then taken out, rinsed with copious DI-water and ethanol, dried at 60 ºC overnight, and directly employed to trigger the anodic reaction of water splitting without carbonization treatment.
